# The duodenal mucosa associated microbiome, visceral sensory function, immune activation and psychological comorbidities in functional gastrointestinal disorders with and without self-reported non-celiac wheat sensitivity

**DOI:** 10.1080/19490976.2022.2132078

**Published:** 2022-10-27

**Authors:** Ayesha Shah, Seungha Kang, Nicholas J Talley, Anh Do, Marjorie M Walker, Erin R Shanahan, Natasha A Koloski, Michael P Jones, Simon Keely, Mark Morrison, Gerald J Holtmann

**Affiliations:** a Department of Gastroenterology and Hepatology, Princess Alexandra Hospital, Brisbane, Australia; b Translational Research Institute Queensland, Australia; c Faculty of Medicine, University of Queensland, Brisbane, Australia; d University of Queensland Diamantina Institute, Woolloongabba, Australia; e College of Health, Medicine and Wellbeing, University of Newcastle, Callaghan, and Hunter Medical Research Institute, New Lambton Heights, Australia; g School of Biomedical Sciences and Pharmacy, University of Newcastle, Callaghan, and Hunter Medical Research Institute, New Lambton Heights, Australia

**Keywords:** Duodenum, mucosa-associated microbiome, functional gastrointestinal disorders, homing small intestinal T-cells, non-celiac wheat sensitivity, self-reported wheat intolerance

## Abstract

Frequently, patients with functional gastrointestinal disorders (FGIDs) report intolerance of wheat products. We compared gastrointestinal symptoms, sensory function, psychiatric comorbidities, gut-homing immune cells, and duodenal mucosa-associated microbiome (d-MAM) in FGID patients and controls with and without self-reported wheat sensitivity (SR-NCWS). We recruited 40 FGID patients and 20 controls referred by GPs for treatment. Gastrointestinal/extraintestinal symptoms, visceral sensory function, psychological comorbidities, and SR-NCWS were assessed in a standardized approach. Peripheral gut homing T-cells (CD4^+^α4^+^β7^+^CCR9^+^/CD8^+^α4^+^β7^+^CCR9^+^) were quantified, and the d-MAM was assessed by DNA sequencing for 46 subjects. Factors of bacterial genera were extracted utilizing factor analysis with varimax rotation and factors univariately associated with FGID or SR-NCWS included in a subsequent multivariate analysis of variance to identify statistically independent discriminators. Anxiety scores (p < .05) and increased symptom responses to a nutrient challenge (p < .05) were univariately associated with FGID. Gut homing T-cells were increased in FGID patients with SR-NCWS compared to other groups (p all <0.05). MANOVA revealed that anxiety (p = .03), visceral sensory function (p = 0.007), and a d-MAM factor comprise members of the *Alloprevotella, Prevotella, Peptostreptococcus, Leptotrichia*, and *Veillonella* lineages were significantly (p = .001) associated with FGID, while gut homing CD4^+^α4^+^ β7^+^CCR9^+^ T-cells were associated (p = .002) with SR-NCWS. Compared to controls, patients with and without SR-NCWS show that there are shifts in the amplicon sequence variants within specific bacterial genera between the FGID subgroups (particularly *Prevotella* and *Streptococcus*) as well as distinct bacterial taxa discriminatory for the two different FGID subtypes. Compared to controls, both FGID patients with and without SR-NCWS have an increased symptom response to a standardized nutrient challenge and increased anxiety scores. The FGID patients with SR-NCWS – as compared to FGID without SR-NCWS (and controls without SR-NCWS) – have increased gut homing T-cells. The d-MAM profiles suggest species and strain-based variations between the two FGID subtypes and in comparison to controls.

## Introduction

Patients presenting with chronic or relapsing gastrointestinal symptoms that are not explained by structural or biochemical abnormalities as the cause of symptoms are referred to as patients with functional gastrointestinal disorders (FGID).^1,2^ Utilizing the Rome Criteria, these patients are categorized based upon their gastrointestinal symptoms into discrete disorders such as irritable bowel syndrome (IBS) or functional dyspepsia (FD).^1^ However, the heterogeneity of these conditions with regard to symptoms and potential triggers of symptoms suggests that within these disorders, there are distinct sub-clinical pathophysiologies.^2^ In recent years, it has been recognized that a considerable proportion of these patients with FGID report symptoms that are triggered or aggravated by the consumption of wheat products and that symptoms improve when wheat containing foods are avoided, even though celiac disease has been excluded. These FGID patients with intolerance of wheat products – without celiac disease as the cause of symptoms – are now frequently referred to as patients with self-reported non-celiac wheat sensitivity (SR-NCWS).^3^

Many patients with FGID have psychiatric comorbidities such as depression and anxiety,^4^ and studies have revealed altered visceral sensory function^5^ and altered immune function with increased circulating gut homing small intestinal T-cells and cytokine release both in IBS and FD.^6^ Furthermore, in recent years, multiple studies point toward associations between FGID and alterations of the gastrointestinal microbiome.^7–12^ Links between the gastrointestinal microbiome and anxiety and depression, potentially mediated by inflammatory pathways, have also been postulated^13^ and demonstrated in animal models.^14^

Symptoms triggered by the consumption of wheat products may change dietary intake of wheat and gluten products, and previous data suggest that a low gluten diet alters the gastrointestinal (stool) microbiome.^15^ Thus, it is important to compare differences in the microbiome between subjects with and without wheat-related symptoms. Recent work has also highlighted the possibility that specific wheat proteins such as amylase trypsin inhibitors can cause immune activation.^16^ Indeed, immune activation is now well recognized in FGID patients, but the link with SR-NCWS remains to be studied.

Previously, we have shown in FGID patients that alterations in the duodenal mucosa-associated microbiome (d-MAM), symptoms, and meal-related quality of life scores are linked.^17^ However, the interrelationships between visceral sensory function, psychological comorbidities, immune activation, SR-NCWS, and the d-MAM remain poorly understood in FGID.^18^

We hypothesize that FGID patients as compared to controls have an augmented symptom response to a standardized nutrient challenge (reflecting visceral hypersensitivity and/or disordered upper gastrointestinal motor function), and an altered d-MAM. In addition, FGID patients with and without SR-NCWS differ regarding immune activation and the d-MAM.

## Results

### Structural lesions in patients with symptoms and controls and self-reported non celiac wheat sensitivity

**Figure 1.**
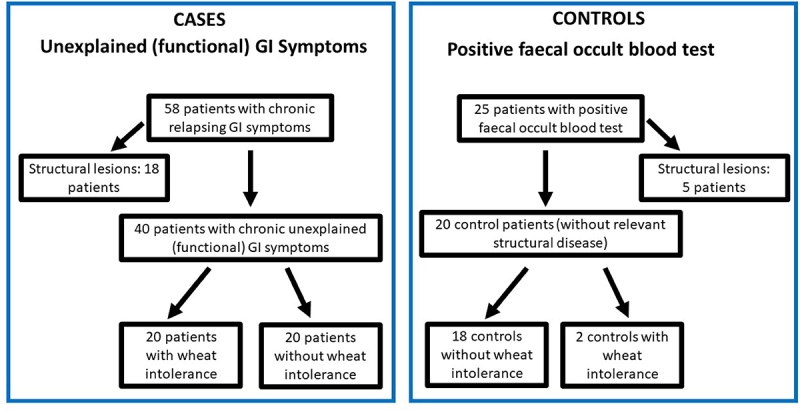
Patients and controls included into the study

Eighteen patients with chronic relapsing symptoms had structural lesions that were potential causes for their gastrointestinal symptoms (six with erosions in the duodenal bulb, two with erosive esophagitis, two with large hiatal hernia, two with gastric ulcer, and one with duodenal ulcer). These patients were excluded from further analyses. In 5/25 controls with a positive FOBT, structural lesions (2 with erosive esophagitis, 1 with colorectal cancer, 1 with inflammatory bowel disease, and 2 with diverticular disease) were identified. These patients were also excluded from the study. Thus, 40 patients with chronic or relapsing unexplained (functional) gastrointestinal symptoms and 20 controls were included. Of the FGID cohort, 20/40 patients with unexplained (functional) gastrointestinal symptoms and 2/20 control subjects (referred for diagnostic work-up of a positive FOBT) reported that the consumption of wheat products induced or aggravated gastrointestinal symptoms and symptom improvement occurred after reduction of wheat consumption (i.e., SR-NCWS; Figure 1).

### Clinical features of FGID and control patients

All 40 patients with unexplained (functional) gastrointestinal symptoms met the Rome IV criteria for IBS and/or FD, while no control met the Rome IV criteria. 30/40 FGID patients had an overlap of FD/IBS.

The characteristics of the different groups are depicted in Table 1. Patients with FGID had significantly higher SAGIS and GIS scores compared to controls (p < .01, Table 1). Similarly, these patients had a significantly higher cumulated symptom score in response to the standardized nutrient challenge compared to controls (p < .01, Table 1). Scores for anxiety (p ≤ .05) or depression (p = .1) as measured with the HADS were higher in FGID patients as compared to controls (Table 1). No differences between FGID patients and controls were found in relation to BMI, smoking habits, or use of medications (statins, non-steroidal anti-inflammatory drugs [NSAID]) but proton pump inhibitor (PPI) use was increased in FGID patients, Table 1).

**Table 1.** Characteristics of FGID patients vs. controls.

	FGID patients (*n* = 40)	Control patients (*n* = 20)	*p*-Value
Age^a^	51.7 (±15.0)	59.5 (±10.8)	0.072
Gender (female), *n* (%)	22 (55.0)	11(55.0)	0.61
BMI^a^	26.9 (±6.1)	29.3 (±8.6)	0.25
Active smoker, *n* (%)	11 (27.5)	4 (20)	0.83
PPI use, *n* (%)	23 (57.5)	2 (10)	**0.004**
***Gastrointestinal symptoms***			
Total SAGIS score^a^	30.4 (±16.4)	2.9 (±3.9)	**<0.001**
SAGIS, epigastric^a^	12.5 (±6.3)	1.1 (±2.0)	**<0.001**
SAGIS, constipation^a^	2.3 (±2.4)	0.4 (±1.0)	**<0.002**
SAGIS, diarrhea^a^	7.3 (±5.1)	0.8 (±1.1)	**<0.002**
SAGIS, nausea and vomiting^a^	4.6 (±3.9)	0.4 (±1.1)	**<0.001**
SAGIS, reflux and dysphagia^a^	3.8 (±2.7)	0.1 (±0.5)	**<0.001**
GIS, score^a^	15 (±8.6)	1.5 (±2.8)	**<0.001**
GI symptoms related to wheat consumption, *n* (%, 95% CI)	20 (50, 95% CI 35–65)	2 (10, 95% CI 0–23)	**<0.05**
***Depression and anxiety scores***			
HADS, anxiety^a^	6.4 (±4.9)	2.6 (±2.4)	**<0.05**
HADS, depression^a^	6.8 (±4.6)	3.6 (±2.2)	0.06
***Gastrointestinal function testing***			
Symptom response to nutrient challenge, score^a^	499 (±469)	132 (±103)	**0.03**

*n*: number; HADS: Hospital Anxiety and Depression Scale; GI: gastrointestinal; GIS: Gastrointestinal symptom score; SAGIS: Structured Assessment of Gastrointestinal Symptoms; PPI: proton pump inhibitor; NSAIDs: non-steroidal anti-inflammatory drugs; BMI: body mass index. ^a^Values expressed as mean (±standard deviation). All the *p* values in bold indicate a statistically significant result.

### Clinical features of FGID patients stratified by self-reported non-celiac wheat sensitivity vs. controls (without self-reported non-celiac wheat sensitivity)

No differences between FGID patients with and without SR-NCWS regarding age, gender, BMI, nicotine consumption, or consumption of PPI or NSAIDs were found (Table 2). There were no significant differences between FGID patients with and without SR-NCWS intolerance in relation to symptom severity by SAGIS (p > .3) or mean scores for anxiety and depression as measured with the HADS (p > .6). Utilizing Rome-IV criteria FGID patients with and without SR-NCWS also indicated similar frequencies of the various FD subtypes and IBS-subtypes (Table 2).

**Table 2.** Characteristics of FGID patients with and without SR-NCWS vs. controls without SR-NCWS.

	FGID patients		Controls without SR-NCWS	Overall *p*-value, FGID vs. controls
	With SR-NCWS (*n* = 20)	Without SR-NCWS (*n* = 20)	(*n* = 18)	
Age^a^	53.8 (±15.0)	49.6 (±15.1)	60.0 (±11.2)	0.16
Gender, female, *n* (%)	12 (60)	10 (50)	10 (56)	0.94
BMI^a^	28.4 (±6.2)	25.3 (±5.7)	30.6 (±8.1)	0.4
Active smoker, %	7 (35)	4 (20)	5 (27.8)	0.7
PPI therapy, *n* (%)	9 (45)	12 (60)	2 (10)	<0.01
***Gastrointestinal symptom pattern***				
Total SAGIS score^a^	30.5 (±20.2) *	30.3(±11.9) *	2.7 (±3.7)	<0.001
- Epigastric^a^	13.0 (±8.1) *	12.1 (±3.7) *	1.1 (±2.1)	<0.001
- Nausea/vomiting^a^	4.7 (±4.6) *	4.5 (±3.2) *	3.8 (±1.1)	<0.001
- Constipation^a^	2.2 (±2.7) *	2.5 (±2.0) *	0.4 (±1.0)	<0.01
- Diarrhea^a^	3.5 (±4.0) *	4.7 (±3.4) *	0.5 (±0.9)	<0.001
NDI total^a^	93.8(±37.3) *^	59.3 (±34.9) *	13.3 (±12.0)	<0.001
***Clinical dyspepsia symptom categories***				
No dyspeptic symptoms, *n* (%)	4 (20)	2 (10)	17 (94.4)	<0.01, 0.44^
Only EPS present, *n* (%)	0	3(15)	0	
Only PDS present, *n* (%)	2 (10)	1(5)	0	
EPS and PDS present, *n* (%)	14 (70)	14 (70)	1 (5.6)	
***IBS-categories***				
No bowel symptoms, *n* (%)	7 (35)	3 (15)	17 (94.4)	0.001, 0.261^
IBS-D, *n* (%)	5 (25)	5 (25)	0	
IBS-C, *n* (%)	2 (10)	2 (10)	0	
IBS-M, *n* (%)	6 (30)	10 (50)	1(5.6)	
***Depression and anxiety scores***				
HADS, anxiety	6.8 (±5.1)	6.1 (±5.0)	2.8 (±2.5)	
HADS, depression	7.4 (±4.6)	6.2 (±4.7)	3.3 (±2.3)	
***Gastrointestinal function testing***				
Nutrient challenge, symptom score	451(±453) *	548 (±495) *	127(±110)	<0.02
***Immune function***				
Gut homing CD4^+^ α4^+^β7^+^CCR9^+^ T-cells^+^	1.4 (±1.2) *	0.4 (±0.3)	0.5 (±0.4)	<0.005, 0.03^
Gut homing CD8^+^ α4^+^β7^+^CCR9^+^ T-cells^+^	0.54 (±0.68) *	0.17 (±0.23)	0.13 (±0.09)	0.04, <0.05^
***Mucosal immune cells***				
Eosinophils/hpf^a^	24.1 (±13.2)	21.1 (±12.5)	28.6 (±15.9)	>0.3, 0.5^
Intraepithelial lymphocytes/hpf ^a^	13.6 (±7.3)	13.5 (±5.4)	15.3 (±7.0)	>0.8, 0.9^

FGIDS: functional gastrointestinal disorders; n: number; %: percentage; BMI: body mass index; HADS: Hospital Anxiety and Depression Scale; SAGIS: Structured Assessment of Gastrointestinal Symptoms; NDI: Nepean Dyspepsia Index; EPS: epigastric pain syndrome; PDS: post prandial distress syndrome; IBS: irritable bowel syndrome; hpf: high power field; TNF-α: tumor necrosis factor α. ^a^ Values expressed as mean (±standard deviation), *p < 0.05 vs. controls; ^ *p* values comparing FGID patients with and without SR-NCWS.

### Gastrointestinal function

**Figure 2.**
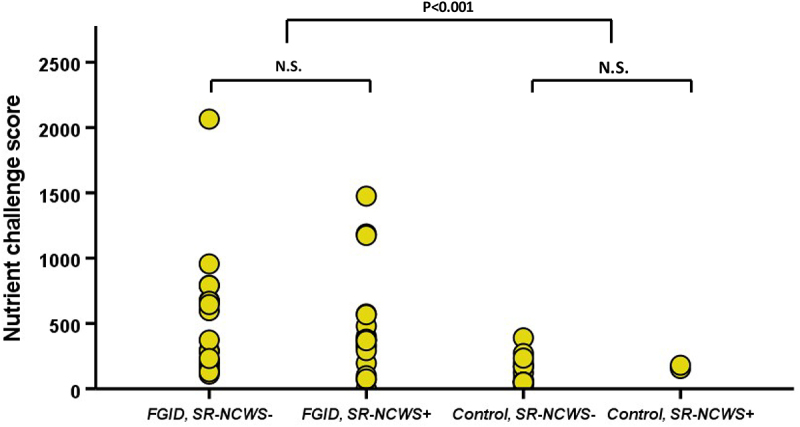
Symptom response to a standardized nutrient challenge in patients with functional gastrointestinal disorders or controls with and without SR-NCWS (P < .001 for FGID vs. Controls, Man-Whitney U-Test.

The symptom response to the standardized nutrient challenge was significantly higher in patients with FGID as compared to the control group (499 ± 469 vs. 132 ± 104, p < .001, Figure 2) but not different for FGID patients with and without SR-NCWS (p > .2).

### Immune function

**Figure 3.**
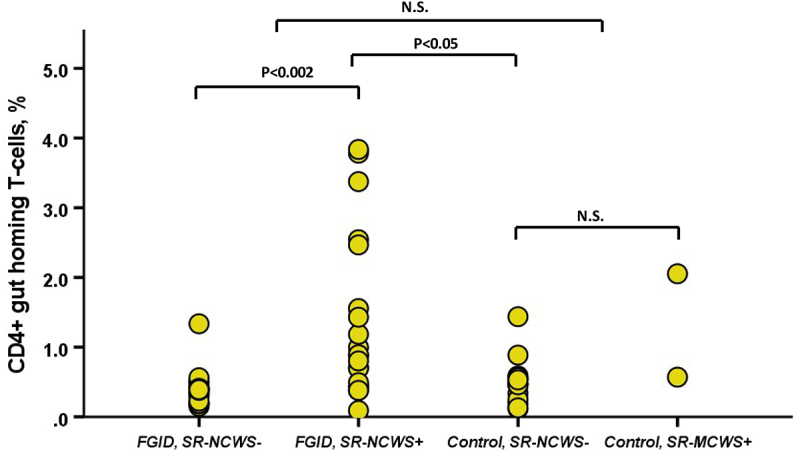
CD4^+^α4^+^β7^+^CCR9^+^ gut homing T-cells in patients with functional gastrointestinal disorders (FGID) or controls with and without SR-NCWS (SR-NCWS- or SR-NCWS+), (P < .002 for FGID, SR-NCWS- vs. FGID, SR-NCWS+ and p < .05 for FGID, SR-NCWS+ vs. Control, NCWS-).

In patients with FGID, the proportion of gut homing CD4^+^α4^+^β7^+^CCR9^+^ and CD8^+^α4^+^β7^+^CCR9^+^ T-cells was significantly higher (p all <0.05) in subjects with SR-NCWS as compared to subjects without SR-NCWS (Figure 3, Table 2). The difference between FGID and controls failed statistical significance (p < .3, Figure 3).

### Psychometric scores and gastrointestinal symptoms, gastrointestinal function, and immune function

The anxiety and depression scores were significantly associated with the total symptom gastrointestinal burden as measured by SAGIS (r = 0.37 and r = 0.51, all p < .01). Similarly, anxiety and depression were statistically significantly correlated with the SAGIS extraintestinal symptom score (r = 0.53 and r = 0.67, all p < .002). In contrast, anxiety and depression were not significantly associated with visceral sensory or gut homing T-cells (p all >0.4).

### Immune activation and gut function

There was a statistically significant positive correlation between the percentage of CD4^+^ α4^+^β7^+^CCR9^+^ gut homing T-cells and the gastrointestinal symptom response to the nutrient challenge (r = 0.43, p < .01), while the association with CD8^+^α4^+^β7^+^CCR9^+^ gut homing T-cells and the symptom response failed statistical significance (r = 0.17, p < .2).

### Duodenal mucosa-associated microbiota (d-MAM)

**Figure 4.**
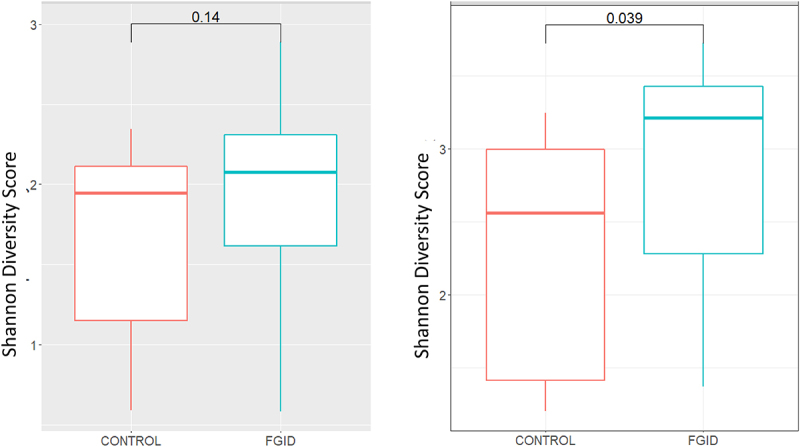
The Shannon diversity scores for the duodenal mucosa-associated microbiota profiles of the control and FGID subjects, at the genus (left) and amplicon sequence variant (ASV “species”, right) levels of classification. Note the trend for increased Shannon diversity among the FGID subjects at the genus level further increases and is statistically significant at the ASV-level, suggesting an expansion in species-diversity within the key genera represented in both subject groups. The boxes represents the boundaries of the first and third quartiles for each group, and the median value is denoted by the internal horizontal line. Whiskers extend to 1.5 times the interquartile range. Pairwise comparisons were made using the Wilcoxon rank-sum test and the calculated p values are shown.

**Figure 5.**
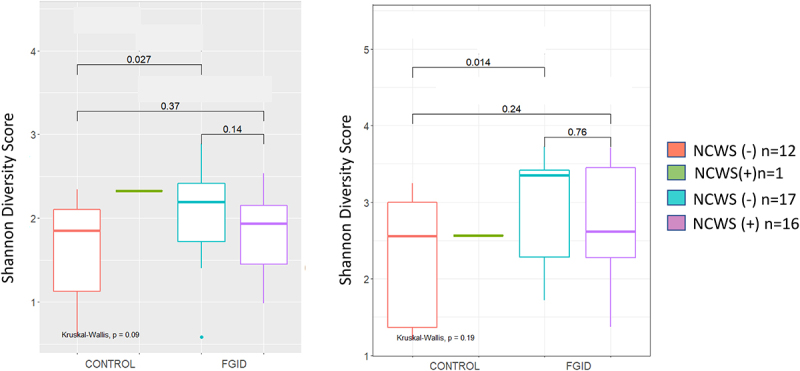
The Shannon diversity scores at the genus (left) and ASV (“species”, right) levels of classification for the duodenal mucosa-associated microbiota of the control and FGID groups, subdivided further into those subjects either reporting (+) or not reporting (-) non-celiac wheat sensitivity (NCWS). Comparisons were first made using the Kruskal–Wallis test and were not significant. However, pairwise comparisons using Wilcoxon rank sum testing showed there is a significant increase in Shannon diversity between the Control and those FGID subjects without NCWS (p = .027 and 0.014, for the genus and ASV-levels, respectively), while the measures for the FGID subjects with NCWS were intermediate to both groups. These results suggest genus/ASV expansion in the FGID group without NCWS but some reduction in diversity in those FGID subjects with NCWS. Boxes represent the boundaries of the first and third quartiles, with the median value is denoted by the internal horizontal line. Whiskers extend to 1.5 times the interquartile range.

The microbiome datasets from 46 patients: 13 Controls (12/13 NCWS (-)); 17 FGID_NCWS (-) and 16 FGID_NCWS (+) passed the quality checks described in the Methods and were used for the subsequent analysis. The composite dataset from the 46 subjects represents 73 genus-level taxonomic groups and 254 amplicon sequence variants (ASVs). Interestingly, while the genus-level Shannon and Simpson measures of within-sample (alpha) diversity were not significantly different between the control and FGID groups, similar analysis at the ASV (“species”) level of classification was both significantly increased for the FGID group (Figure 4 and Figure S2, for Shannon and Simpson measures, respectively). When the microbiome data from the control and FGID groups were then separated into those with or without SR-NCWS, the differences among the subgroups were not significant via Kruskal–Wallis testing. However, the pairwise comparisons by Wilcoxon rank sum tests showed that the Shannon (Figure 5) and Simpson (Figure S3) diversity measures were significantly increased at both the genus and ASV (“species”) level of classification between the control and FGID subgroups without SR-NCWS. Interestingly, there was a small but not significant reduction in both these diversity scores for the FGID patients with SR-NCWS compared to those without SR-NCWS, which resulted in there also being no significant differences between this subgroup and the control subjects (Figure 5 and Figure S3). We next assessed whether the differences in the alpha diversity measures between the two FGID subgroups might be attributable to the use (or nonuse) of proton pump inhibitors (PPI). These results are shown in Figure S4. At both the genus- and ASV-levels of classification, both Kruskal-Wallis and Wilcoxon tests suggest that there were no significant differences between the PPI users and nonusers with or without SR-NCWS. We next used the TSS normalized data at the ASV-level for PCoA analysis of the beta (between sample) phylogeny-based (weighted and unweighted UniFrac) and compositional dissimilarity (Bray-Curtis) metrics, and the results are shown in Figures S5, S6, and S7, respectively. Although there was a separation of a small number of the PPI nonusers from the rest of the study participants, the analyses suggest there was no significant clustering based on either subject classification, NCWS status, or PPI use. Nor were any significant differences predicted for these metrics by ADONIS permutation testing. In summary, our findings suggest that there is an expansion of the “species” diversity within the major bacterial taxa in the d-MAM of FGID subjects compared to controls, although the magnitude of this change appears to be attenuated in the FGID subjects with SR-NCWS. Additionally, PPI use (or nonuse) does not appear to exert substantive changes on the alpha- and beta-diversity measures of the d-MAM used here.

**Figure 6.**
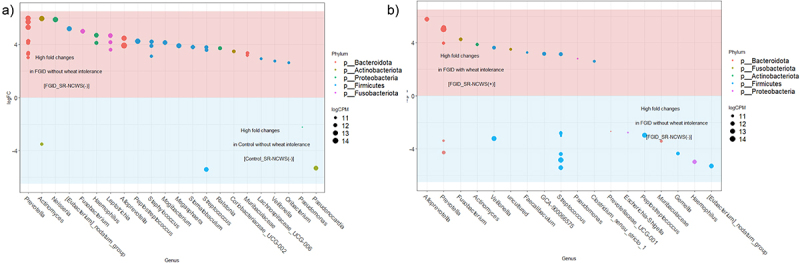
Genus-level and amplicon sequence variant (ASV) differentiation of the duodenal mucosa-associated microbiota between the FGID and control subjects without self-reported non-celiac wheat sensitivity (SR-NCWS, panel A); and between the FGID patients only, either with or without NCWS (panel B). Each datum point represents an ASV (“species”) assigned to the genera listed along the x axis and considered to be significantly different between the groups being compared (p < .01 and FDR <0.01). Each ASV is color coded according to its phylum-level classification, and its size represents the log2 counts per million. In panel A, the relative abundance of 40 ASVs representing 22 genera were different between the FGID and control patients without SR-NCWS. In panel B, the relative abundance of 30 ASV (“species”) representing 18 genera were different between the FGID patients with or without NCWS. These ASV variations suggest there may be “species-specific” variations that differentiate between the subject classification groups. A more detailed tabulated version of the analyses presented in panels A and B are provided as Supplementary Tables 1 and 2, respectively.

To attempt to examine and identify these “species-level” differences, we next performed a differential abundance analysis using edgeR, which showed 40 ASVs assigned to 22 different genera differentiated between the control and FGID patients without SR-NCWS (Figure 6a and corresponding Table S1). At the levels of difference used (P < .01 and FDR <0.01) only the abundances of single ASV assigned to each of the genera *Actinomyces, Streptococcus*, and *Pseudonocardia* were greater in the control subjects without SR-NCWS. In contrast, multiple ASV assigned to genus *Prevotella* (8), Leptotrichia and Staphylococcus (3), Neisseria Haemophilus, and Alloprevotella (2), and two ASV assigned to the family *Muribaculaceae* accounted for ~50% of the differences for FGID patients without SR-NCWS. The *edgeR* analysis also provided further differentiations between the FGID subjects with or without SR-NCWS (Figure 6b and corresponding Table S2). Of the 30 ASV representing 18 different genera, there was a more even distribution of the number of taxa differentiating between the two subgroups and, particularly, differences in the ASV profiles for the genus *Prevotella* and *Streptococcus* ASV. Interestingly, the FGID subjects with SR-NCWS are differentiated by several poorly characterized or “uncultured” taxa, whereas the FGID subjects without SR-NCWS possess ASVs assigned to additional key asaccharolytic and proteolytic bacteria such as *Veillonella, Peptostreptococcus*, and *[Eubacterium]nodentum* group.

**Figure 7.**
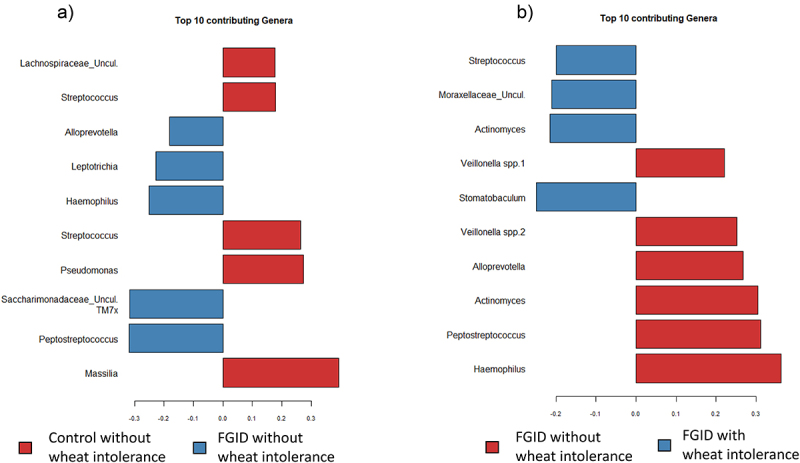
The bacterial ASV (“species”) identified by sparse Partial Least Squares Discriminant Analysis (sPLS-DA) to be discriminatory of the duodenal mucosa-associated microbiota from: A) the FGID (blue) and control subjects (red) without self-reported non-celiac wheat sensitivity; and B) between the FGID patients only, either with (blue) or without self-reported non-celiac wheat sensitivity (red). Each panel shows the top 10 ASVs contributing to the variance captured by the first component in the sPLS-DA model via mixOmics.

A constrained sparse Partial Least Squares-Discriminant Analysis (sPLS-DA) was also performed, and the top 10 discriminatory ASV of the control and FGID subgroups without SR-NCWS and between the FGID subgroups with and without SR-NCWS are shown in Figures 7a and 7b, respectively. These analyses showed that, like the *edgeR* analysis, ASV assigned to the genus *Alloprevotella, Leptotrichia, Haemophilus*, and *Peptostreptococcus* were discriminatory for the FGID patients without SR-NCWS; and in a similar vein that *Streptococcus* was discriminatory of the control subjects without SR-NCWS (Figure 7a). The sPLS-DA modeling of differences between FGID patients with or without SR-NCWS shows that, like the *edgeR* analysis, ASV assigned to *Veillonella, Haemophilus*, and *Peptostreptococcus* was discriminatory for the FGID patients without SR-NCWS, and a single ASV from the genus *Streptococcus* and *Actinomyces* was discriminatory for FGID patients with SR-NCWS.

### Factor analysis

Finally, we used factor analysis of the genus-level relative abundance data to identify eight genera grouped into two common factors, indicating correlations between the abundance values of the component genera (Table 3). Of these, only factor 1 which is comprised of *Alloprevotella, Prevotella, Peptostreptococcus, Veillonella, and Leptotrichia*, approached a statistically significant association with FGID (p < .09, Table 4). Multivariate ANOVA revealed that anxiety and the symptom response to the nutrient challenge were both independently associated with FGID, anxiety, nutrient challenge, and the first genus factor all independently discriminated FGID vs controls, whereas gut homing CD4+ α4+ β7+ CCR9+ and CD8 cells were independently associated with SR-NCWS (Table 5 and Table 6).

**Table 3.** Loadings of genera onto common factors. Eight genera were grouped into two common factors, indicating correlations between the abundance values of the component genera. Scores derived from the composite genera are then used in Table 4.

Factor	Genus	Loading
1	*Veillionella*	0.70
	*Prevotella*	0.64
	*Leptotrichia*	0.62
	*Alloprevotella*	0.62
	*Peptostreptococcus*	0.58
2	*Faecalibaculum*	0.72
	*Gemella*	0.50
	*Streptococcus*	−0.31

**Table 4.** Distribution of microbiome genus factor scores and other factors in FGID vs. controls.

*Genus factor*	Controls, mean (SD)	FGID, mean (SD)	*p*-Value
1	−0.40 (0.43)	0.16 (0.96)	0.09
2	0.11 (1.26)	−0.04 (0.47)	0.32
***Other factors***			
CD4^+^ gut homing	0.70 (0.65)	0.94 (1.05)	0.62
CD8^+^ gut homing	0.18 (0.17)	0.38 (0.58)	0.59
Visceral sensory function	139.75 (111.67)	486.94 (490.45)	0.008
HADS anxiety	2.57 (2.37)	6.12 (5.27)	0.14
HADS depression	3.57 (2.23)	6.32 (4.55)	0.17

HADS: Hospital Anxiety and Depression Scale; FGID: functional gastrointestinal disorders; SD: standard deviation.

**Table 5.** Distribution of genus factor scores and other factors in wheat-related symptoms.

*Genus factor*	No symptoms, mean (SD)	Wheat symptoms, mean (SD)	*p*-Value
1	0.02 (0.96)	−0.04 (0.72)	0.78
2	−0.07 (0.84)	0.12 (0.62)	0.17
*Other factors*			
CD4^+^ gut homing	0.46 (0.34)	1.50 (1.24)	<0.001
CD8^+^ gut homing	0.16 (0.20)	0.58 (0.71)	0.02
Visceral sensory function	353.92 (439.79)	422.27 (452.62)	0.81
HADS anxiety	5.10 (4.81)	5.75 (4.45)	0.89
HADS depression	5.50 (4.42)	6.08 (4.21)	0.65

HADS: Hospital Anxiety and Depression Scale; SD: standard deviation.

**Table 6.** Multivariate findings, discriminator of (a) FGID vs. controls and (b) wheat symptoms vs. none.

Discriminator	Effect	p-Value
***a) FGID vs. controls***		
d-MAM Factor 1	0.80 (0.34, 1.26)	0.001
Nutrient challenge score	340.72 (93.57, 587.87)	0.007
HADS anxiety	3.38 (0.43, 6.33)	0.03
***b) Wheat symptoms vs. no wheat symptoms***		
CD4	1.17 (0.42, 1.93)	0.002
CD8	0.42 (0.01, 0.82)	0.047

Based on multivariate ANOVA model that included both genus factors, CD4+, CD8+, nutrient challenge text, and HADS, statistical inference via nonparametric bootstrap.

## Discussion

This is the first clinical study that has assessed the symptom response to a standardized nutrient challenge (as a measure of visceral sensory function and motor function), activation of gut-homing T-cells, psychological comorbidities, and the d-MAM, in FGID patients and controls with and without SR-NCWS. A significant difference in the symptom-response to a standardized nutrient challenge and psychological comorbidities differentiates FGID patients and controls, while no difference in relation to visceral sensory function or psychological comorbidities between FGID patients with and without SR-NCWS are found. However, gut homing T-cells (CD4^+^α4^+^β7^+^CCR9^+^/CD8^+^α4^+^β7^+^CCR9^+^) were more than twofold higher in FGID patients (and controls) with SR-NCWS when compared to subjects without SR-NCWS, suggesting a role of immune activation for the manifestation of SR-NCWS. Furthermore, distinct microbial profiles are observed for subjects with and without SR-NCWS, while there are also differences between FGID patients and controls. Taken together, the data point toward multifactorial disease mechanism(s) causing FGID and SR-NCWS. While visceral sensory function and psychological comorbidities are common features of FGID patients, immune activation appears to be associated with SR-NCWS and with d-MAM differences across both groups.

Intolerance of wheat products is increasingly recognized in subjects with FGID,^19,20^ and it has been argued that they present a distinct group of patients. In our study, 50% of FGID patients had SR-NCWS. Interestingly, in previous studies, the prevalence of SR-NCWS in the population was approximately 15%,^21^ while the prevalence of FD and IBS in the population was approximately 36%.^22^ Patients with SR-NCWS frequently complain of FGID symptoms such as abdominal pain or discomfort, bloating, and diarrhea,^23^ suggesting a link between FGID and SR-NCWS. Given the absence of any biomarkers to identify the agent causing the symptoms and the impracticality of performing dietary elimination followed by double-blind placebo-controlled challenges in daily clinical practice, this entity is now addressed as “self-reported wheat sensitivity (SR-WS).”^24^ Furthermore, clinical trials have questioned that gluten is responsible for symptoms in FGID patients because symptoms were not aggravated after exposure to gluten^25^ in patients with SR-NCWS. In contrast, other wheat ingredients including fructans (belonging to fermentable oligo-, di-, and monosaccharides and polyols [FODMAPs]) have been considered as possible factors for symptom generation/exacerbation.^25^ For instance, there is evidence that increased fermentable carbohydrate exposure^26^ – in a hypersensitive intestine^27^ – can induce symptoms.^28^ In addition, there is cumulating evidence that specific wheat proteins such as Amylase Trypsin Inhibitors (ATIs) induce intestinal immune activation.^29^ Thus, wheat ATIs are now considered as a potential causal factor for the manifestation of wheat intolerance.^30^ The wheat ATIs are typically protease resistant and activate the toll-like receptor 4 (TLR4) complex in the monocytes, macrophages, and dendritic cells of the intestinal mucosa.^31^ With oral ingestion, they can co-stimulate antigen presenting cells and promote T-cell activation not only in celiac disease but also in other immune-mediated diseases within and outside the gastrointestinal tract.^32^ These findings are also well aligned with a previous study reporting^33^ atypical food allergies in more than 50% of IBS patients. This study utilized confocal laser endomicroscopy for real-time detection and quantification of changes in intestinal tissues and cells, including fluid extravasation in response to food antigens applied as solutions/suspensions of wheat, milk, soy, yeast, or egg white. In this study, classical food allergies had been ruled out by negative results from immunoglobulin E serology and skin testing. Still, more than 60% of patients had visible changes of the duodenal mucosa after exposure to wheat extract utilizing confocal laser endomicroscopy. This finding is consistent with the increased gut homing CD4^+^α4^+^β7^+^CCR9^+^/CD8^+^ α4^+^β7^+^CCR9^+^ T-cells in patients with SR-NCWS.

There is consideration that SR-NCWS may be linked to the GI microbiome – either directly by facilitating the manifestation of SR-NCWS or by FGID subjects consciously or unconsciously altering the food intake with subsequent effects on the gastrointestinal microbiome. Here, we found that the intra-sample (alpha diversity) metrics were reduced at the genus level, and significantly so at the ASV (“species”) level for the control group compared to the FGID group. Additionally, the FGID subgroup comparisons showed there was a decrease in these metrics for those FGID patients with SR-NCWS, but still remained greater than that found for the control group. While the available data for the d-MAM are relatively limited, recent studies have reported that the alpha diversity of duodenal aspirates is reduced for FGID patients.^34^ We note here that Shannon and Simpson diversity metrics at the genus level of classification show similar trends to those reported by Wauters et al.^35^ who reported that these metrics were similar (at the genus level) between the healthy control and “FD-starters” in samples of duodenal epithelium and mucus. However, our results presented here suggest that there is an expansion of the differences between the control and FGID groups at the ASV-level of microbe classification, similar to our related unpublished studies using a larger cohort of control and FGID subjects (Shanahan et al., in review). In that context, we also note that while studies of the luminal and/or stool microbiota frequently report reduction in alpha-diversity metrics that are concordant with microbial “dysbiosis” in subjects, there are also exceptions, such as the microbial dysbiosis of female urogenital tract during pregnancy associated with preterm births and overweight/obesity.^36,37^ As such and going forward, the clinical definition of “control subjects” and/or collection methods of samples from the proximal small intestine need careful consideration.^38,39^

Although we have previously shown that PPI-use can influence the gastrointestinal microbiome via increased bacterial load on duodenal tissue,^7^ our results suggest that PPI use or nonuse appears to have no profound impacts on the alpha- or beta-diversity measures for the d-MAM of the control and FGID patients with or without SR-NCWS. These findings are also consistent with the recent studies by Wauters et al.^35^ and Weitsman et al.^40^ who found some genus- and family-level differences in the microbiota between PPI-users and PPI nonusers but no major shifts in the mucosa-associated or duodenal aspirate profiles associated with this intervention.

In this study, we have compared all the FGID subjects with the controls using three distinct statistical approaches: *edgeR*, sPLS-DA (ASV-level), and factor analysis (genus-level). One or more of these approaches identified specific ASVs representing *Prevotella, Alloprevotella, Neisseria, Veillonella, Peptostreptococcus*, and *Leptotrichia* to be increased for the FGID patients compared to the control group. These differences are likely to support a mixed acid fermentation including succinate and propionate production, which in turn provides substrates that can support the growth of asaccharolytic *Veillonella* spp. and *Neisseria spp.*,^41^ respectively. As such, these alterations in the d-MAM profiles of FGID patients without SR-NCWS are microbiologically intuitive and aligned with other findings. Indeed, a previous study focussing on IBS patients observed in the small intestine significantly increased the abundance of *Prevotella* spp, and *Prevotella* and *Veillonella* spp. abundance was significantly correlated.^42^

Interestingly, the FGID subgroup comparisons by *edgeR* identified many of the differences were intra-genus variations of *Prevotella, Alloprevotella, Veillonella*, and *Streptococcus* ASVs. In contrast, ASV assigned to the genus *Veillonella*, and the genera *Gemella, Peptostreptococcus, Staphylococcus*, and *Neisseria* were significantly greater in FGID patients without SR-NCWS, and similar findings were made by sPLS-DA. Wheat sensitivity is deemed to be attributable to immunogenic peptides and/or proteins such as gluten and more recently amylase-trypsin inhibitors (ATI). Indeed, a recent animal study revealed that at least one strain of *Veillonella* spp. (R39-8) can degrade at least some ATI-variants, the destruction of which was shown to reduce their inflammatory effects in the rodent animal model.^43^ Perhaps then, the presence of specific *Veillonella* spp., as well as *Peptostreptococcus* and *Gemella*, has a protective proteolytic potential that mitigates the effects of ATI consumed with wheat products. In that context, the increase in gut homing T-cells in subjects with SR-NCWS might be a reflection of strain-dependent changes in the proteolytic potential of the d-MAM toward ATIs. Thus, our results suggest that there is an ecological drift in the d-MAM profiles of FGID patients with SR-NCWS characterized by intra-genus (species-level) variations of *Prevotella, Streptococcus*, and *Veillonella* lineages, which may explain why antimicrobial therapies (e.g., with rifaximin, a non-absorbable antibiotic) are efficacious in a subset of patients with FGID.^44^ While the findings for the d-MAM profiles are based on associations and thus need to be interpreted with caution, they reveal key bacterial taxa that should be tested with *ex-vivo* approaches combining microbe- and tissue cell-culture from clinical specimens to further elucidate potential effects in relation to mucosal immune activation.^45^

Previous studies have consistently reported that in a subgroup of FGID patients circulating gut homing small intestinal T-cells (α4β7 integrin) are increased.^2,46–48^ Our study now reveals that the increase in circulating gut homing small intestinal T-cells (α4β7 integrin) may occur predominantly in patients with SR-NCWS. Thus, there is a significant difference between FGID patients with and without SR-NCWS in relation to gut homing small intestinal T-cells. Interestingly, this is consistent with other studies who described small intestinal immune activation in patients with NCWS as reflected by an increase in mucosal immune cells.^49^ On the other hand, the difference between FGID patients and controls without SR-NCWS just failed statistical significance (p = .11) while in a previous study^48^ the comparison of IBS patients and controls revealed a significant difference. The previous study focussed on IBS patients and the larger control population were asymptomatic subjects without any gastrointestinal symptoms and without SR-NCWs. All these points toward the concept that in FGID patients with SR-NCWS an activation of the mucosal immune system occurs, and it might be speculated that the immune activation observed in FGID patients is triggered by specific components in addition to gluten, such as ATIs that are found in wheat and similar grains. However, the multivariate analysis model revealed that one of the four microbiome factors generated by factor analysis, as well as sensory function and anxiety differentiated FGID patients from controls, while immune activation differentiated subjects with and without SR-NCWS. Thus, while FGID and SR-NCWS frequently overlap, immune activation seems likely to be a key feature of SR-NCWS. On the other hand, FGID – irrespective of SR-NCWS – are linked to psychiatric comorbidities and altered sensory function. Thus, the common denominator for FGID (with and without SR-NCWS) is the altered brain gut interaction. This is well aligned with the current concept of FGID as disorders are associated with psychiatric comorbidities such as anxiety and depression^50^ or altered visceral sensory function^5^ as a reflection of disturbed brain–gut interactions.^51^

Our study is not without limitations. The study participants were all recruited via a single center, and we acknowledge that the observational and cross-sectional nature of the study has inherent limitations and that the number of subjects used in the current study is modest. Furthermore, we recruited as controls ‘asymptomatic’ subjects who participated in a colon cancer screening program and had a positive FOBT instead of ‘healthy volunteers.’ The benefit, however, that outweighs this limitation is the fact that all subjects underwent comprehensive diagnostic work-up, and relevant structural lesions potentially causing symptoms were excluded. In addition, subjects with NCWS were identified based upon a structured interview assessing symptoms during and after wheat consumption instead of utilizing a standardized gluten challenge. However, we consider that the detailed clinical workup of the subjects and our use of several different approaches for the (micro)biological comparisons provides novel insights into the host–microbe interactions in the upper small intestine with FGID and SR-NCWS. In that context, we assessed as a marker for visceral sensory function the symptom response to a standardized nutrient challenge with a liquid meal that contained defined amounts of fat, proteins, and carbohydrates. Historically, it is believed that impaired gastric accommodation contributes to the manifestations of meal related (dyspeptic) symptoms^52^ and that delayed gastric emptying plays a role in the pathophysiology of FGID.^53^ Thus, the increased symptom response to the standardized nutrient challenge is not simply a reflection of visceral sensitivity but might also be influenced by reduced gastric accommodation or delayed gastric emptying.^54^ Indeed, in a subset of our larger cohort of control and FGID subjects where gastric emptying has also been measured, we have observed a negative association between gastric emptying time and the relative abundance of the genus *Veillonella^55^* (Shanahan et al., in press). On the other hand, medications that have been shown to reduce postprandial gastric volume (and thus impair postprandial gastric accommodation) such as itopride^56^ have been found to be effective for symptom improvement in patients with functional dyspepsia.^57^ Similarly, while delayed gastric emptying is thought to be linked to symptoms, acceleration of gastric emptying with a potent prokinetic neither in patients with normal nor in patients with delayed gastric emptying has closely correlated with improvement of symptoms.^58^ This suggests delayed gastric emptying or impaired postprandial gastric relaxation are markers but not the causes of symptoms. However, we have shown in the past as part of the validation and routine use of our nutrient challenge tests^59^ that the symptom response to the test meal is correlated with sensory thresholds as measured with a barostat.^60^ All this suggests that the differences in the symptom response to a standardized nutrient challenge reflect visceral sensory function albeit modified by alterations of gastric emptying or fundic relaxation. Against this background, the nutrient challenge is considered a minimally invasive and accepted surrogate marker for visceral sensory function.^61^

Another limitation is that we did not strictly use the Salerno criteria^62^ with the proposed double-blinded gluten challenge to confirm NCWS. Based upon our experience, only a limited number of patients would comply with several weeks of dietary restrictions and would be prepared to have a standardized gluten or wheat exposure. This would result in a highly selected patient cohort. Instead, patients were interviewed in relation to symptoms occurring when wheat products were consumed. All patients with SR-NCWS reported that within 7 days of regular ingestion of wheat products, gastrointestinal and/or extraintestinal symptoms manifested, and these symptoms improved or even disappeared within a week after discontinuation or marked reduction of wheat consumption. We did not assess the severity of symptoms during exposure (and symptom improvement after discontinuation of wheat consumption) since this is likely a function of dose and duration of exposure which were not standardized. In a large population-based study, the prevalence of SR-NCWS was 14% and linked to FGID, female gender, and younger age.^63^ On the other hand, a very elegant study from Norway^64^ demonstrated that symptoms in subjects with SR-NCWS were linked to the consumption of fructans but not gluten. Thus, SR-NCWS may not identify subjects with intolerance of gluten but other dietary components. Indeed, our data suggest that the simple clinical categorization based upon self-reported wheat symptoms is of relevance. Furthermore, all our study subjects underwent comprehensive diagnostic work-up to capture a multitude of factors potentially contributing to their symptoms. Thus, there is a risk that we may have recruited patients with more severe symptoms when compared to typical FGID patients seen in the primary care setting. However, when symptom severity of our patient cohort as measured by the validated SAGIS instrument was compared to a cohort of nearly 2,000 consecutive patients referred for the management of chronic unexplained (functional) symptoms, their symptom intensities were similar to this large reference population of patients seen at a tertiary hospital (Figure S1). This gives confidence that our patients are representative of the FGID patients who are seen in the secondary or tertiary health-care setting.

In *summary*, relative to control subjects without symptoms, FGID patients had a significantly increased symptom response to a standardized nutrient challenge, increased burden from psychological comorbidities, and alterations of the d-MAM favoring ASV- (“species”) level shifts toward lineages of *Prevotella, Alloprevotella, Neisseria, Peptostreptococcus* and *Leptotrichia*. In FGID-patients with SR-NCWS, gut-homing T-cells were increased as compared to FGID (and controls) without SR-NCWS, and further ASV (“species”)-level shifts, particularly within the *Prevotella* and *Streptococcus* genera, were discriminatory between the FGID patients with or without SR-NCWS. These findings suggest that the proteolytic capabilities of the d-MAM should be further characterized regarding their *ex-* and *in vivo* capabilities to modulate functions that are potentially associated with FGID and the manifestation of SR-NCWS.

## Methods

We recruited 83 patients (58 with chronic or relapsing gastrointestinal symptoms meeting Rome IV criteria^65^ for IBS and/or FD and 25 controls) referred for the assessment of iron deficiency (with or without anemia) or a positive focal occult blood test (FOBT). All FGID patients (FD and/or IBS) were referred by their general practitioners for diagnostic work-up, and management after empiric treatments had failed to achieve sufficient control of gastrointestinal symptoms. All patients with FGID had undergone standard treatment including *H. pylori* testing and eradication if positive, treatment with proton pump inhibitor (PPI) if dyspeptic symptoms were present, or dietary interventions including a low FODMAP diet, or other dietary interventions had been trialed as clinically appropriate. Since these treatments did not provide sufficient symptom improvement, they had been discontinued and at the time of inclusion into the study, all patients were on a normal western diet for at least 8 weeks prior to enrollment.

All subjects underwent comprehensive routine clinical assessment and outpatient work-up as deemed clinically appropriate by the treating physician including anti-tissue transglutaminase immunoglobulin A (IgA) antibodies, measurement of total serum IgA levels while on a gluten rich diet to rule out celiac disease and patients had normal duodenal biopsies (Marsh 0) performed at the time of endoscopy while on a gluten-containing diet. All controls were required to have no or minimal gastrointestinal symptoms (all SAGIS scores ≤1) and negative endoscopic findings with the exception of polyps that were removed during the colonoscopy.

### Assessment of gastrointestinal symptoms

The presence and severity of gastrointestinal and extraintestinal symptoms, bowel habits, and psychiatric comorbidities were clinically assessed and categorized. Consistent with the Rome IV^65^ criteria, patients were clinically categorized as FD, IBS, or FD/IBS overlap. In addition, patients were categorized as having epigastric pain syndrome (EPS), postprandial distress syndrome (PDS), or diarrhea or constipation predominant IBS (IBS-D, IBS-C) or mixed IBS (IBS-M). Furthermore, symptom severity and psychosocial factors were assessed utilizing validated questionnaires: the Structured Assessment of Gastrointestinal Symptoms (SAGIS)^66^ questionnaire and the Gastrointestinal Symptom Score (GIS),^67^ the Hospital Anxiety and Depression Scale (HADS),^68,69^ and the Nepean Dyspepsia Index (NDI).^70^ All patients recruited into this study underwent a standardized interview by a study nurse to determine if symptoms occurred in relation to the consumption of wheat products and had improved after discontinuation or reduction of wheat consumption. Based upon the responses, patients were categorized as patients with self-reported wheat symptoms. SR-NCWS was diagnosed when the patient reported during a standardized interview that within 7 days of regular ingestion of wheat products gastrointestinal and/or extraintestinal symptoms manifested, and these symptoms improved or even disappeared within a week after discontinuation or reduction of wheat consumptions.

### Categorization of study participants

Patients meeting the inclusion criteria (FGID: Rome IV, controls: FOBT+ without relevant gastrointestinal symptoms and without clinically significant findings on endoscopy) were categorized as FGID patients with and without SR-NCWS and Controls with and without SR-NCWS (Figure 1).

### Endoscopic procedures

Patients primarily underwent gastroscopy and colonoscopy following routine procedures. For all the procedures, standard Olympus equipment (Olympus EVIS EXERA III, CV-190, Olympus irrigation pump and Olympus processor, Tokyo, Japan) were used. During endoscopy, routine biopsies were taken as clinically indicated. In addition, four intestinal biopsies were taken with the Brisbane Aseptic Biopsy device (MTW, North Rhine Westphalia, Germany), from the second part of the duodenum and further processed utilizing the previously described aseptic techniques.^7,71^

### Histology

At endoscopy, standardized biopsies for histology and microbial analyses were taken from the second portion of duodenum (D2). For duodenal pathology, the architecture of the villi, intraepithelial lymphocyte (IEL) counts, and presence and grade of acute and chronic inflammation were recorded. Eosinophil counts in five non-overlapping high-power fields (HPF) were performed in the second part of duodenum (D2) and summed to give a mean total count of 5 HPF.

### Standardized nutrient challenge test (NCT)

The symptom response to a standardized nutrient challenge (as a measure of visceral sensitivity)^61^ was assessed on the day following the completion of the structured assessment of gastrointestinal symptom questionnaire described above. After an 8-hour fast, subjects were asked to drink 200 ml of a standardized nutrient liquid (Ensure®) every 5 minutes up to a cumulative volume of 600 ml. Before and 5 min after each 200 ml drink, symptoms were assessed using a visual analog scale (range 0–100 mm) with 0 = no symptom and 100 = unbearably severe. This tool assesses five symptoms: fullness, abdominal pain, nausea, retrosternal/abdominal burning, and acid regurgitation. The cumulative symptom responses were determined, and the cumulative scores for each symptom individually and for all symptoms combined were used as the primary outcome variables.^5^

### Flow cytometry

Blood samples were taken, and isolated peripheral blood mononuclear cells (PBMCs) were incubated with human BD Fc Block prior to being labeled with optimal concentrations of monoclonal antibodies (Becton-Dickinson, North Ryde, NSW, Australia) directed against human, CD3 (BV510), CD4 (APC-Cy7), CD8 (FITC), CD49d/α4-integrin (PE-CF594), β7-integrin (PE), and CCR9 (Alexa647). Each sample containing 1 × 10^6^ of labeled PBMCs was analyzed using BD LSRFortessa Cell Analyzer with data of 1 × 10^5^ events collected. After excluding dead cells (BD Fixable Viability stain), gut homing T-cell population was defined as CD3^+^CD4^+^ α4^+^ β7^+^CCR9^+^ or CD3^+^CD8^+^ α4^+^ β7^+^CCR9^+^ using Kaluza Analysis software.

### Characterization of the duodenal mucosa-associated microbiota (d-MAM)

The protocols used here for DNA extraction from biopsy tissue and 16S rRNA gene amplicon library construction and sequencing of the d-MAM are described in detail by Shanahan et al.^72^ and the supplementary materials and methods. The methods used for data trimming, quality checks, and construction of the table of amplicon sequence variants (ASVs) are also described in the supplementary materials and methods. Briefly, datasets from subjects that produced >500 reads, after filtering to remove taxa represented at ≤0.05% relative abundance, were included in the analyses via the Phyloseq package in R.^73^

### Clinical data and statistical analyses

In a first step, FGID patients and control subjects and subsequently subjects with and without SR-NCWS were compared with respect to quantitative measures using univariate discrimination of groups, e.g., via the Mann–Whitney test due to non-normal distribution of some quantitative measures. With respect to categorical measures, the Pearson Chi-Square test was used. In addition, the links between the psychological parameters, symptom severity, or links with the relative abundance of specific microbial genera were tested utilizing Spearman rank correlations.

In relation to the microbiome data, the alpha (within-sample) diversity was quantified using both the Shannon and Simpson’s diversity index.^74^ Pairwise comparisons of these metrics were made using Wilcoxon Rank Sum tests when comparing all controls and all FGID subjects. When both these groups were subdivided relatively to the subjects in each group with or without SR-NCWS, the alpha diversity metrics were first compared by the Kruskal Wallis test and subsequent pairwise comparisons (excluding the single control subject with SR-NCWS) using Wilcoxon Rank Sum exact test. Weighted and unweighted UniFrac metrics^75^ and the Bray-Curtis dissimilarity metric were calculated as measures of the beta (between sample) diversity and were assessed by principal coordinate analysis (PCoA). A permutational multivariate analysis of variance (PERMANOVA, R function ADONIS via vegan, 999 permutations)^76,77^ was also performed to compare the differences between the patient groups.^78^ Further group comparisons were made using *edgeR*^79^ package via ‘phyloseq_to_edgeR’ command (i.e., control without SR-NCWS versus FGID without SR-NCWS; and FGID patients with (+) and without (-) SR-NCWS). The ASVs with log_2_ fold-changes (logFC) shown to be statistically significant (*p* < .01) and with a false discovery rate (FDR) <0.01 were retained. Modeling via sparse linear discriminant analysis (sPLS-DA) subsequent to centered-log transformation of the ASV data was performed with the MixOmics package in R^73,80^ with taxa discriminatory for the respective groups relative to the primary component of variance visualized using the ggplot2 graphics package in R.^81^ .

Besides the above-described analyses of the d-MAM, in a separate independent step, the genus-level relative abundance data were also characterized via an exploratory factor model, with factors extracted using principal axis factoring and rotated using varimax rotation to ensure statistically independent factors. The number of factors was determined using a parallel analysis followed by factor with the first *n* factors whose eigenvalues were above chance-expectation selected. Scores for each factor for individual patients were calculated using the regression method.^82^

Subsequently, statistically independent discriminators of groups were determined via multivariate analysis of variance (MANOVA) with all measures identified as univariately statistically significant being considered for the multivariate model. The assumption of multivariate normality was evaluated via the Doornik–Hansen test and was found to be statistically significant (p < .001), indicating a violation of that assumption. For this reason, formal statistical inference employed the nonparametric bootstrap with 1000 iterations.

Quantification of the degree of discrimination was via the area under the receiver-operator characteristic curve formed from predicted probabilities estimated from unconditional logistic models in which statistically independent discriminators identified in the MANOVA step were entered into the model. The model was estimated via multiple imputation due to the pattern of missing values.

Statistical analyses of clinical, immune, and sensory testing and the factor analysis were undertaken utilizing SPSS version 26 (IBM Inc., Armonk, NY, USA) and Stata (StataCorp. 2019. Stata Statistical Software: Release 16. College Station, TX: StataCorp LLC). The microbial statistical analyses were performed by the various R packages as described above. The sample size was limited by patient availability but provides adequate statistical power (>0.8) at the 0.05 level of statistical significance for effect sizes corresponding to Cohen’s d of 0.8 for contrasts of functional with healthy subjects and 0.9 for contrasts of wheat sensitive versus nonsensitive functional subjects.

## Supplementary Material

Supplemental Material

## Study Approval:

The Ethics approval was obtained from the respective Institutional Review Board (Metro South Health and the University of Queensland (Research Protocol HREC/13/QPAH/690)), and all participants provided written informed consent to participate in the study.
