# Supplementary material for: The duodenal mucosa associated microbiome, visceral sensory function, immune activation and psychological comorbidities in functional gastrointestinal disorders with and without self-reported non-celiac wheat sensitivity
Source: Gut Microbes. 2022 Oct 27;14(1):2132078. doi: 10.1080/19490976.2022.2132078 (PMC9621048; doi:10.1080/19490976.2022.2132078)
Supplement: Supplemental Material [file KGMI_A_2132078_SM6178.zip › KGMI_20211017R2_Supplementary_Figures.pdf]

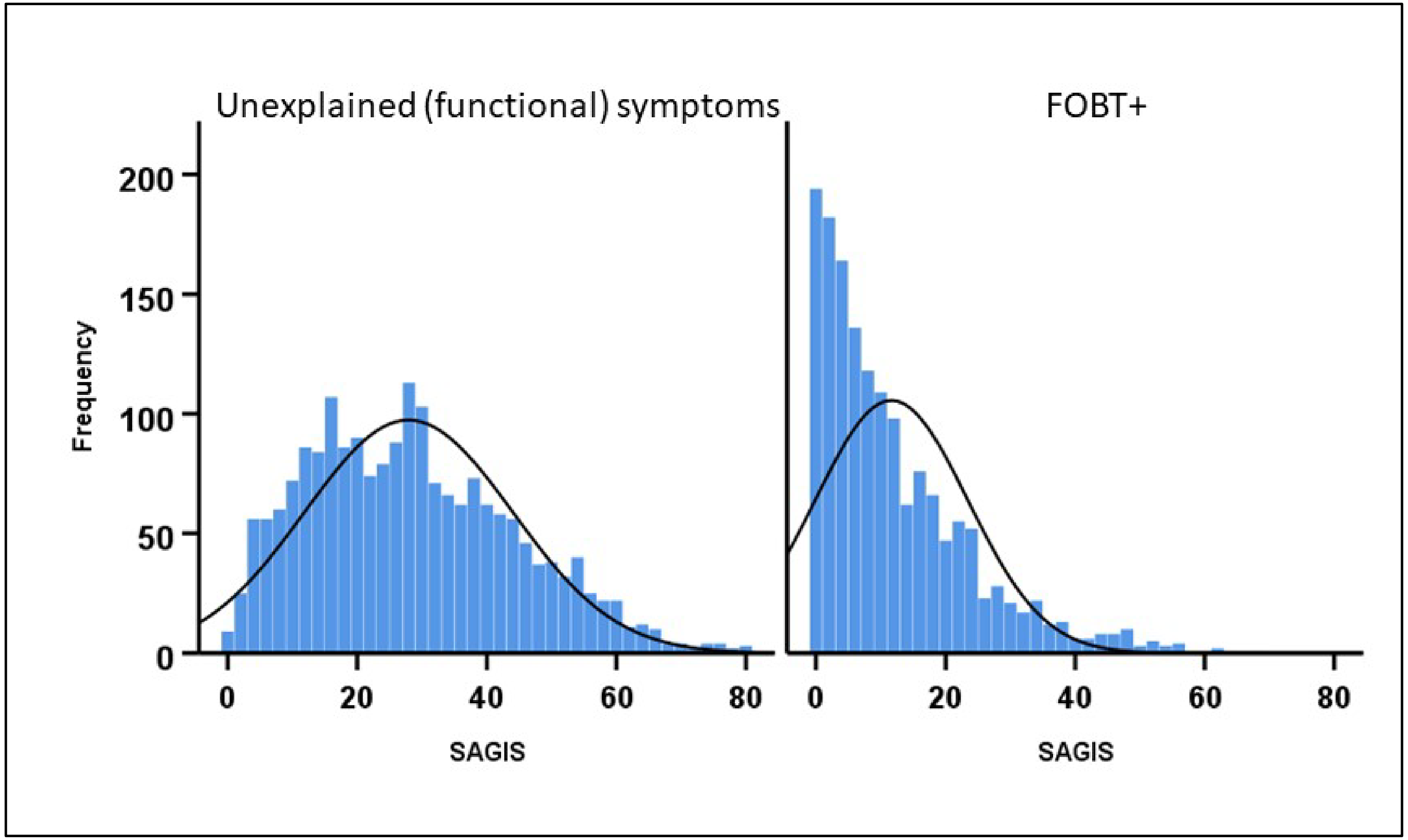

Figure S1: Frequency distribution of SAGIS symptom scores (as assessed during the initial consultation with specialist) in consecutive patients referred by their General Practitioners for assessment and treatment of symptoms or diagnostic work-up of a positive FOBT. In patients labelled 'unexplained (functional) symptoms' the symptoms remained unexplained after comprehensive diagnostic work-up. Mean SAGIS score for patients with unexplained symptoms patients (n=1955) = 27.9 (+16.0) vs. 11.6 (+11.7) in FOBT positive patients (n=1554, p<0.001). In the current study FGID patients had a mean SAGIS score of 30.4(±16.4) as compared to 2.9 (±3.9) in subjects with a positive FOBT (controls).

Figure S2

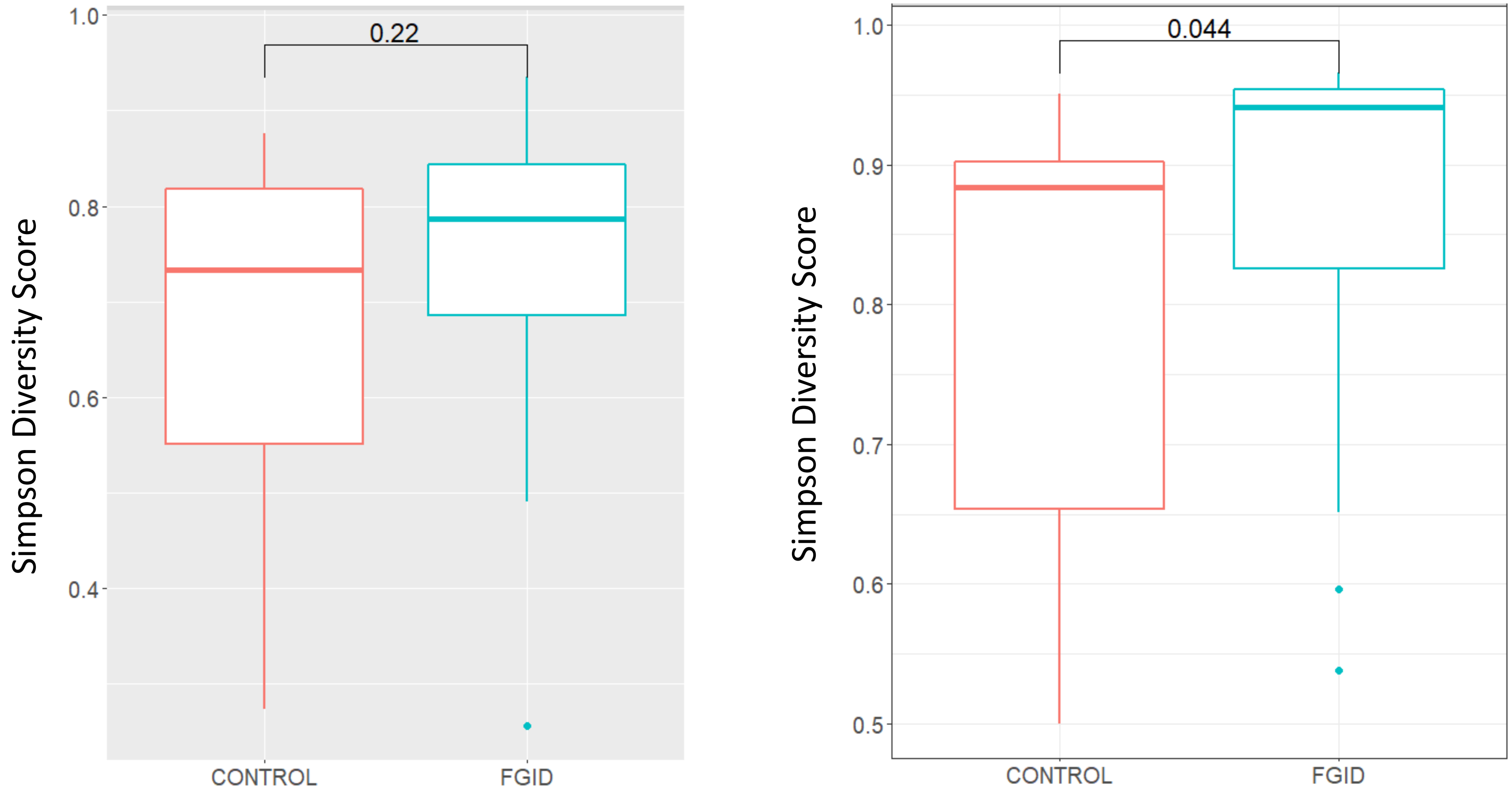

Figure S2: The Simpson diversity scores for the duodenal mucosa-associated microbiota profiles of the control and FGID subjects, at the genus (left) and amplicon sequence variant (ASV "species", right) levels of classification. Note the trend for increased Simpson diversity among the FGID subjects at the genus level further increases and is statistically significant at the ASV-level, suggesting an expansion in species-diversity within the key genera represented in both subject groups. The boxes represents the boundaries of the first and third quartiles for each group, and the median value is denoted by the internal horizontal line. Whiskers extend to 1.5 times the interquartile range. Pairwise comparisons were made using the Wilcoxon rank-sum test and the calculated p values are shown.

Figure S3

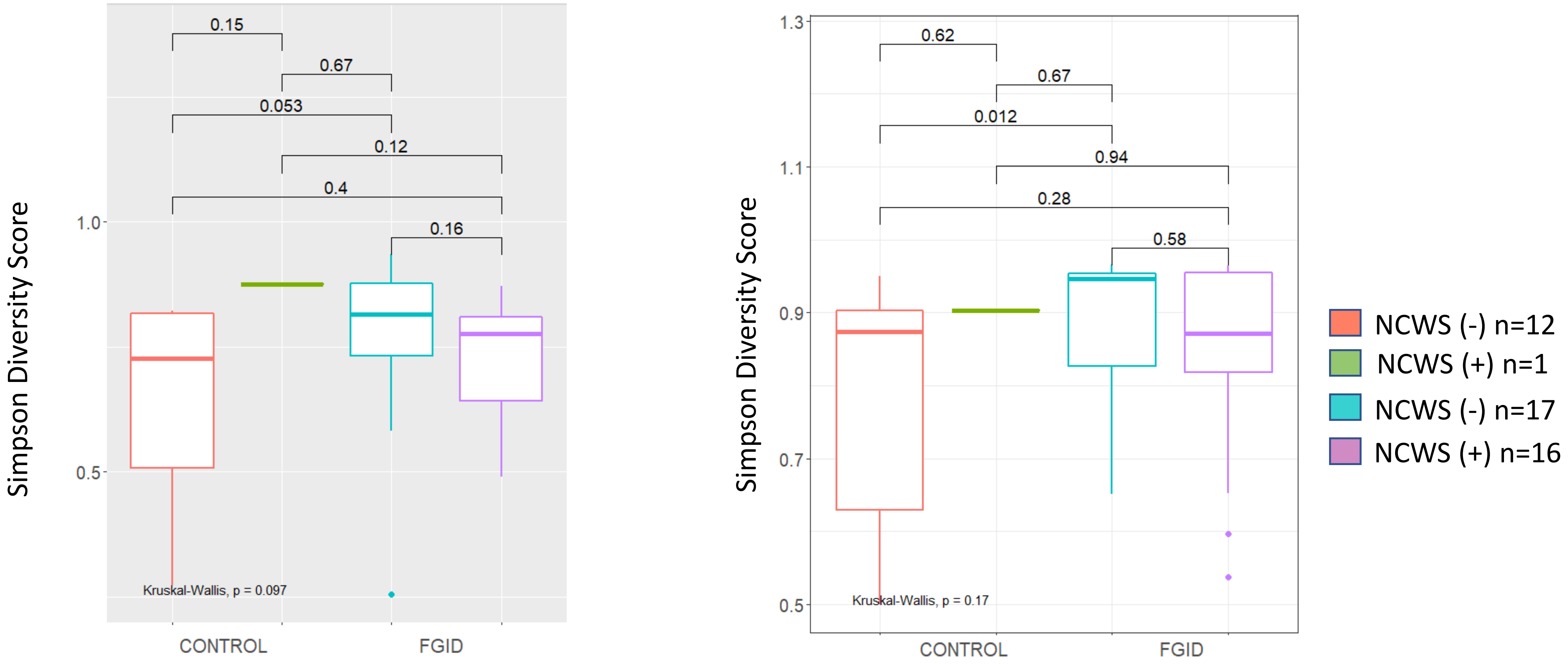

Figure S3: The Simpson diversity scores at the genus (left) and ASV ("species", right) levels of classification for the duodenal mucosa-associated microbiota of the control and FGID groups, subdivided further into those subjects either reporting (+) or not reporting (-) non-celiac wheat sensitivity (NCWS). Comparisons were first made using the Kruskal-Wallis test and were not significant. However, pairwise comparisons using Wilcoxon rank sum testing showed there are increases in the Simpson diversity scores at both genus (p=0.053) and ASV (p=0.012) levels between the Control and those FGID subjects without NCWS; while the measures for the FGID subjects with NCWS were intermediate to both groups. These results suggest genus/ASV expansion in the FGID group without NCWS but some reduction in diversity in those FGID subjects with NCWS. Boxes represent the boundaries of the first and third quartiles, with the median value is denoted by the internal horizontal line. Whiskers extend to 1.5 times the interquartile range.

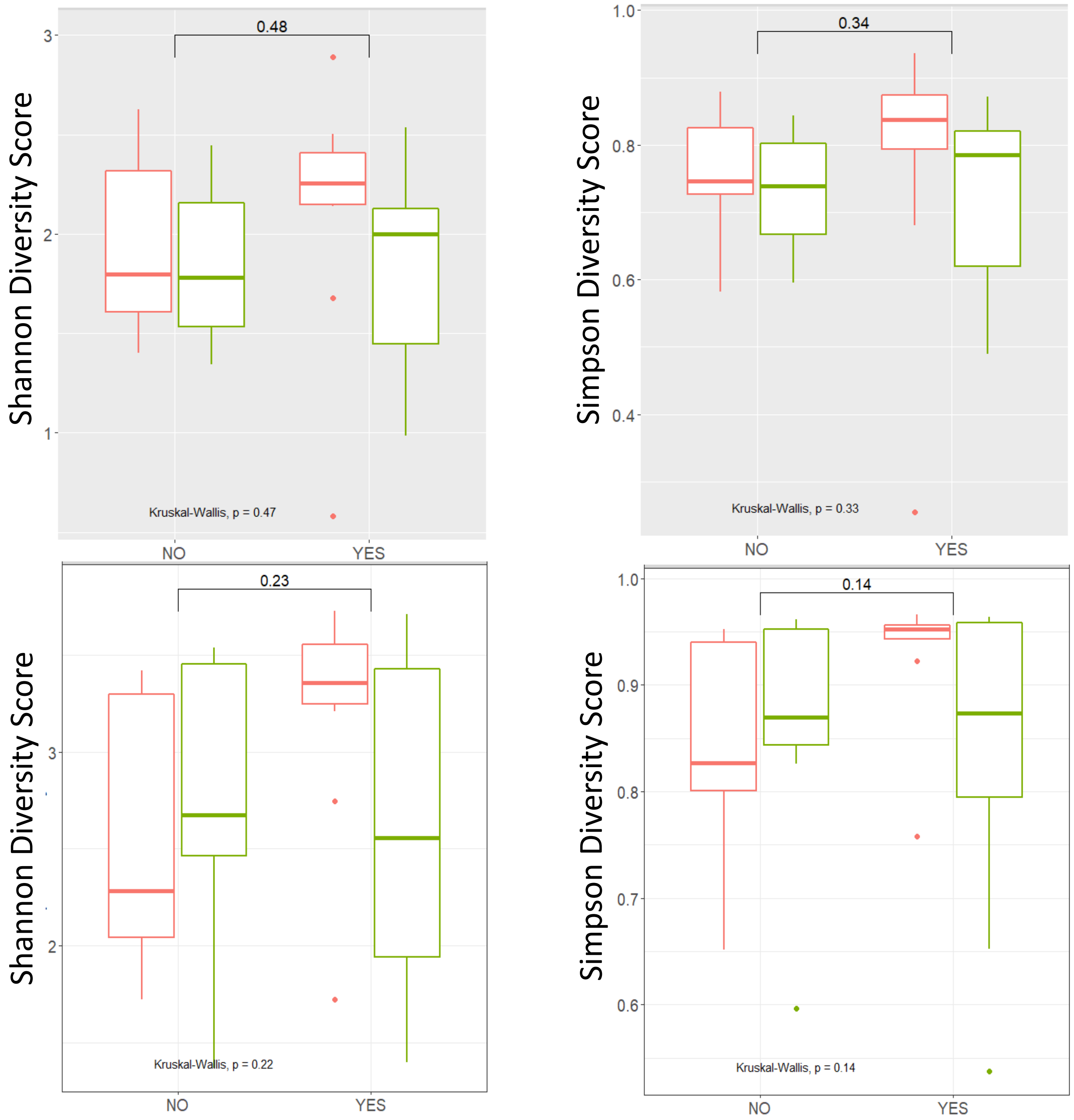

Figure S4: There are no significant differences in the alpha (within sample) Shannon (left) and Simpson (right) diversity measures at the genus (top) and amplicon sequence variant ("species", bottom) of the FGID subjects either without (red) or with non-celiac wheat sensitivity (green); and further subdivided according to PPI non-users (NO, n=15) or PPI users (YES, n=19). The p values calculated using the Kruskal-Wallis test are noted for each and were not significant; nor were the p values for PPI users/non-users as evaluated by Wilcoxon rank sum testing. The box whisker plots are constructed and annotated as described previously.

Figure S5

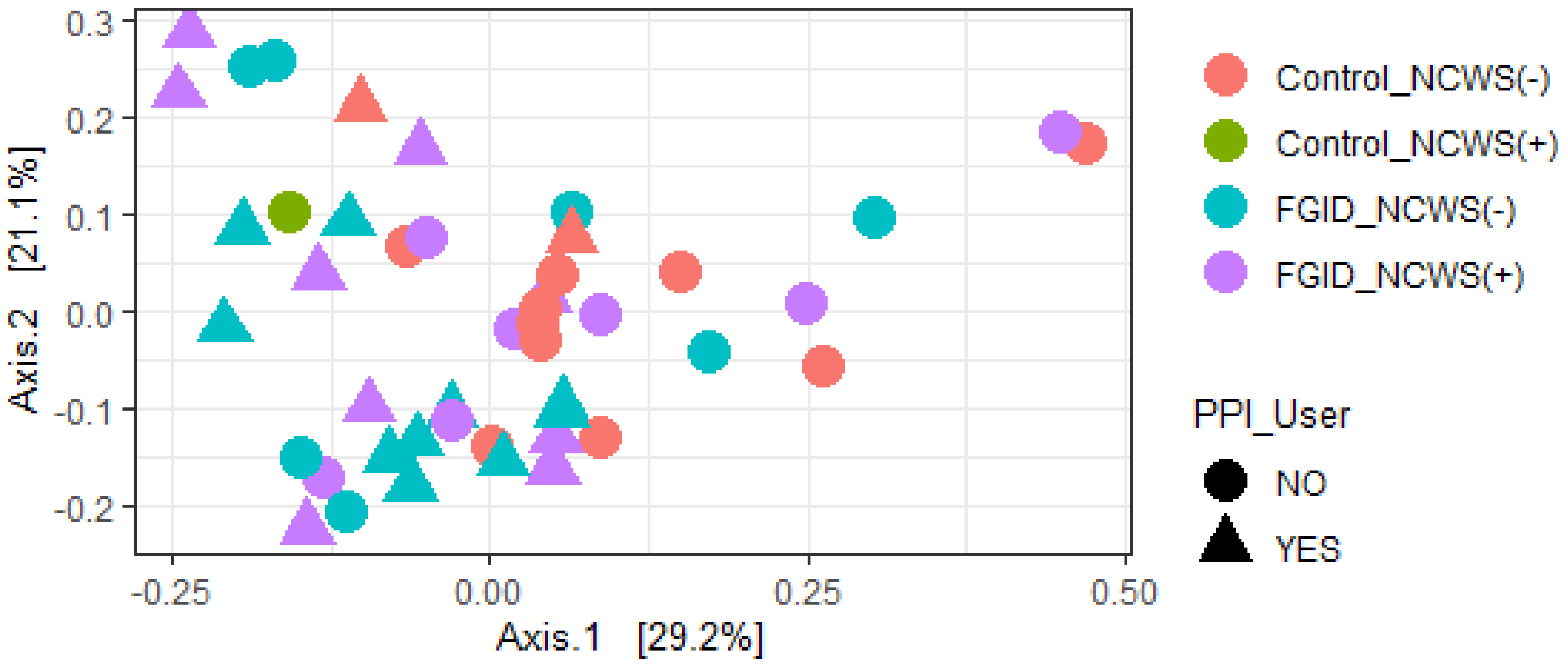

Figure S5: Principal Coordinate Analysis (PCoA) of the weighted Unifrac distance metrics at the ASV-level of classification. The Control and FGID subjects with or without NCWS are color coded, as are PPI users and non-users by the symbols, as described in the key. The results of the ADONIS permutation test were not significant (p = 0.21) and there doesn't appear to be any distinctive clustering and there was no apparent clustering attributable to the patient subgroups and/or between PPI users and non-users.

Figure S6

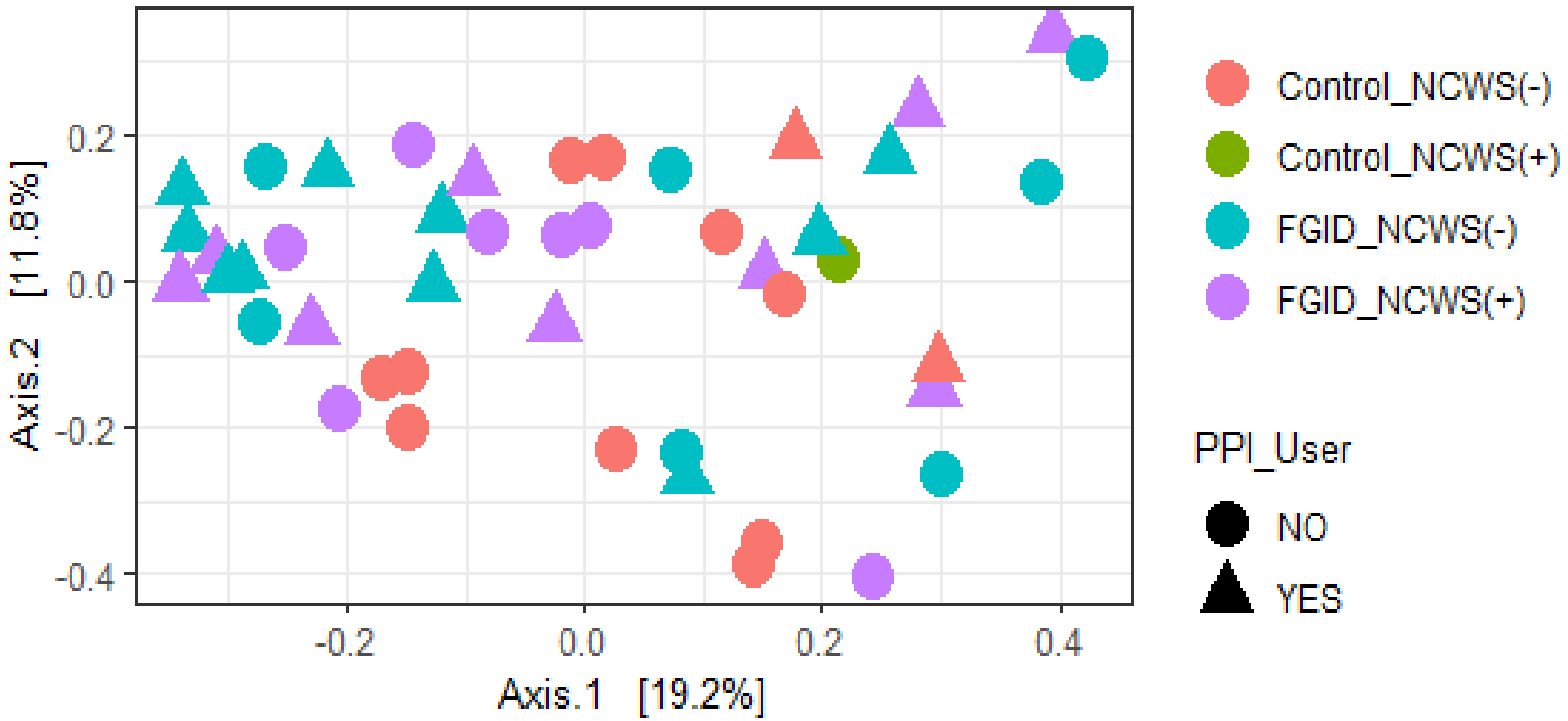

Figure S6: Principal Coordinate Analysis (PCoA) of the unweighted UniFrac distance metrics at the ASV-level of classification. The Control and FGID subjects with or without NCWS are color coded, as are PPI users and non-users, as described in the key. The results of the ADONIS permutation test were not significant (p = 0.11) and there was no apparent clustering attributable to the patient subgroups and/or between the PPI users and non-users.

Figure S7

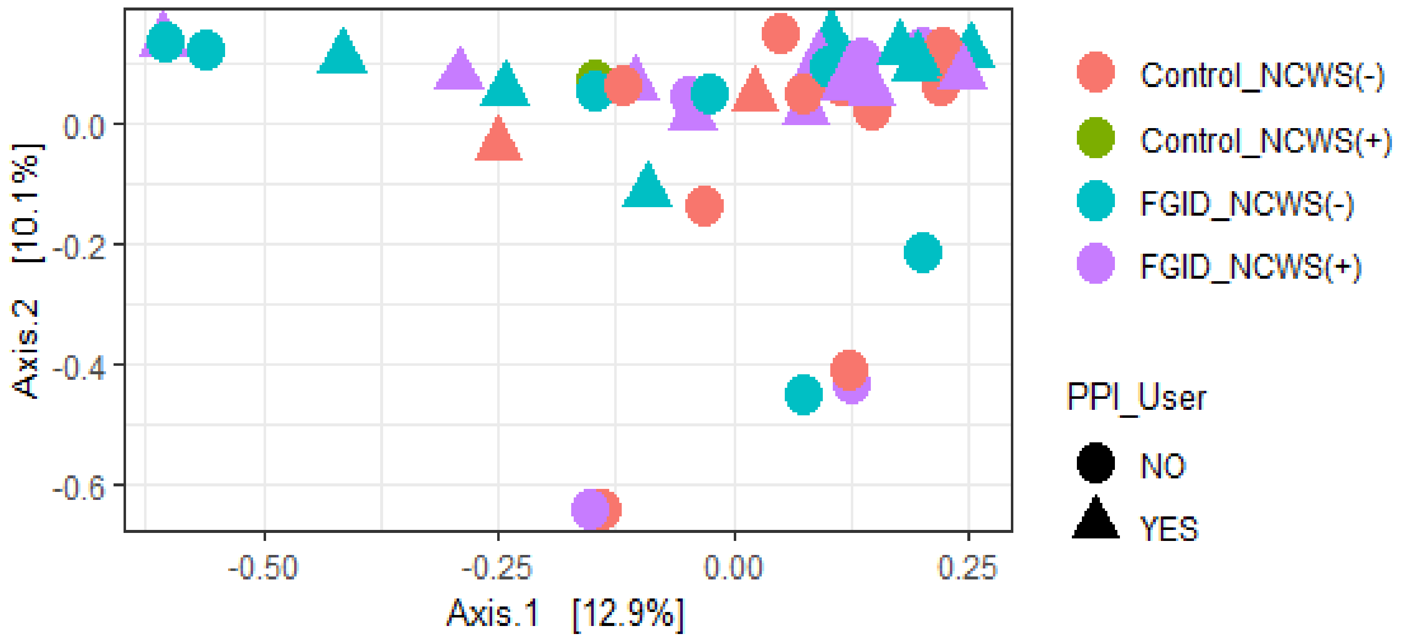

Figure S7: Principal Coordinate Analysis (PCoA) of the Bray Curtis dissimilarity metrics at the ASV-level of classification. The Control and FGID subjects with or without NCWS are color coded, as are PPI users and non-users, as described in the key. The results of the ADONIS permutation test were not significant ($p = 0.33$) and there was no apparent clustering attributable to the patient subgroups and/or between the PPI users and non-users.
