# Supplementary material for: The duodenal mucosa associated microbiome, visceral sensory function, immune activation and psychological comorbidities in functional gastrointestinal disorders with and without self-reported non-celiac wheat sensitivity
Source: Gut Microbes. 2022 Oct 27;14(1):2132078. doi: 10.1080/19490976.2022.2132078 (PMC9621048; doi:10.1080/19490976.2022.2132078)
Supplement: Supplemental Material [file KGMI_A_2132078_SM6178.zip › KGMI_20211017R2_Supplementary_File_CLEAN.docx]

**Supplementary file for manuscript:** **The Duodenal Mucosa Associated Microbiome, Visceral Sensory Function, Immune Activation and Psychological Comorbidities in Functional Gastrointestinal Disorders with and without Self-reported Non-celiac Wheat Sensitivity**

**Legend of Tables**

**Supplementary Table 1:** The Amplicon Sequence Variants (ASV) denoted at the Phylum, Family and Genus-level identified by *edgeR* analysis to be differentially abundant between control and FGID patient groups without self-reported non celiac wheat sensitivity (SR-NCWS) (Figure 4., A). The log2-fold change (logFC), log-counts per million reads (logCPM), P and FDR values for those taxa are also shown. Those genera with negative logFC values were found in greater relative abundances in the control group, positive logFC values represent those taxa found in greater relative abundances in the FGID without self-reported wheat intolerance.

**Supplementary Table 2:** The Amplicon Sequence Variants (ASV) denoted at the Phylum, Family and Genus-level identified by *edgeR* analysis to be differentially abundant between the FGID patients with or without self-reported non celiac wheat sensitivity (SR-NCWS) (Figure 4., B). The log2-fold change (logFC), log-counts per million reads (logCPM), P and FDR values for those taxa are also shown. Those genera with negative logFC values were found in greater relative abundances for FGID patients without SR-NCWS, while the Genus with positive logFC values were found in greater relative abundances in the FGID patients with SR-NCWS.

**Supplementary Table 3:** List of contaminant ASVs generated by the phyloseq “prune_taxa” function. Any ASV observed at a relative abundance greater than 1% on average across 20 reagent controls was included. This criterion ensured all significant contaminants could be removed from the patient samples.

**Supplementary Table 1:** The Amplicon Sequence Variants (ASV) denoted at the Phylum, Family and Genus-level identified by *edgeR* analysis to be differentially abundant between control and FGID patient groups without self-reported non celiac wheat sensitivity (SR-NCWS) (Figure 4., A). The log2-fold change (logFC), log-counts per million reads (logCPM), P and FDR values for those taxa are also shown. Those genera with negative logFC values were found in greater relative abundances in the control group, positive logFC values represent those taxa found in greater relative abundances in the FGID without self-reported wheat intolerance.

| **Phylum** | **Family** | **Genus** | **logFC** | **logCPM** | **PValue** | **FDR** |
| --- | --- | --- | --- | --- | --- | --- |
| Firmicutes | *Streptococcaceae* | *Streptococcus* | -5.412 | 12.653 | 0.000 | 0.000 |
| Actinobacteriota | *Pseudonocardiaceae* | *Pseudonocardia* | -5.325 | 12.573 | 0.000 | 0.000 |
| Actinobacteriota | *Actinomycetaceae* | *Actinomyces* | -3.515 | 11.145 | 0.000 | 0.000 |
| Proteobacteria | *Pseudomonadaceae* | *Pseudomonas* | -2.240 | 10.417 | 0.001 | 0.003 |
| Firmicutes | *Lachnospiraceae* | *Oribacterium* | 2.639 | 10.904 | 0.002 | 0.008 |
| Firmicutes | *Veillonellaceae* | *Veillonella* | 2.767 | 10.987 | 0.002 | 0.007 |
| Firmicutes | *Lachnospiraceae* | *Lachnospiraceae_UCG-006* | 2.939 | 11.105 | 0.000 | 0.002 |
| Bacteroidota | *Prevotellaceae* | *Prevotella* | 3.006 | 11.151 | 0.001 | 0.005 |
| Firmicutes | *Staphylococcaceae* | *Staphylococcus* | 3.116 | 11.229 | 0.001 | 0.006 |
| Bacteroidota | *Muribaculaceae* | *Muribaculaceae* | 3.181 | 11.276 | 0.001 | 0.004 |
| Bacteroidota | *Prevotellaceae* | *Prevotella* | 3.283 | 11.810 | 0.002 | 0.010 |
| Bacteroidota | *Muribaculaceae* | *Muribaculaceae* | 3.347 | 11.398 | 0.000 | 0.002 |
| Bacteroidota | *Prevotellaceae* | *Prevotella* | 3.372 | 11.416 | 0.001 | 0.003 |
| Actinobacteriota | *Atopobiaceae* | *Coriobacteriaceae_UCG-002* | 3.487 | 11.503 | 0.000 | 0.002 |
| Firmicutes | *Streptococcaceae* | *Streptococcus* | 3.574 | 11.570 | 0.000 | 0.002 |
| Fusobacteriota | *Leptotrichiaceae* | *Leptotrichia* | 3.620 | 11.606 | 0.000 | 0.002 |
| Proteobacteria | *Burkholderiaceae* | *Ralstonia* | 3.705 | 12.036 | 0.000 | 0.002 |
| Firmicutes | *Streptococcaceae* | *Streptococcus* | 3.787 | 11.738 | 0.000 | 0.002 |
| Firmicutes | *Lachnospiraceae* | *Stomatobaculum* | 3.805 | 12.398 | 0.000 | 0.002 |
| Firmicutes | *Staphylococcaceae* | *Staphylococcus* | 3.883 | 11.816 | 0.000 | 0.002 |
| Bacteroidota | *Prevotellaceae* | *Alloprevotella* | 3.909 | 14.757 | 0.002 | 0.007 |
| Firmicutes | *Veillonellaceae* | *Megasphaera* | 3.919 | 13.002 | 0.000 | 0.002 |
| Proteobacteria | *Pasteurellaceae* | *Haemophilus* | 4.106 | 11.998 | 0.000 | 0.001 |
| Bacteroidota | *Prevotellaceae* | *Prevotella* | 4.112 | 12.004 | 0.000 | 0.001 |
| Firmicutes | *Anaerovoracaceae* | *Mogibacterium* | 4.147 | 12.553 | 0.000 | 0.001 |
| Fusobacteriota | *Leptotrichiaceae* | *Leptotrichia* | 4.157 | 12.041 | 0.000 | 0.002 |
| Firmicutes | *Staphylococcaceae* | *Staphylococcus* | 4.222 | 12.096 | 0.000 | 0.002 |
| Firmicutes | *Peptostreptococcaceae* | *Peptostreptococcus* | 4.247 | 13.444 | 0.000 | 0.001 |
| Bacteroidota | *Prevotellaceae* | *Prevotella* | 4.251 | 12.120 | 0.000 | 0.001 |
| Bacteroidota | *Prevotellaceae* | *Alloprevotella* | 4.473 | 13.813 | 0.000 | 0.002 |
| Fusobacteriota | *Leptotrichiaceae* | *Leptotrichia* | 4.659 | 12.472 | 0.000 | 0.001 |
| Proteobacteria | *Pasteurellaceae* | *Haemophilus* | 4.686 | 12.496 | 0.000 | 0.000 |
| Fusobacteriota | *Fusobacteriaceae* | *Fusobacterium* | 4.990 | 12.767 | 0.000 | 0.000 |
| Firmicutes | *Anaerovoracaceae* | *[Eubacterium]_nodatum_group* | 5.197 | 12.955 | 0.000 | 0.000 |
| Bacteroidota | *Prevotellaceae* | *Prevotella* | 5.285 | 13.035 | 0.000 | 0.000 |
| Bacteroidota | *Prevotellaceae* | *Prevotella* | 5.297 | 13.517 | 0.000 | 0.001 |
| Bacteroidota | *Prevotellaceae* | *Prevotella* | 5.682 | 13.405 | 0.000 | 0.000 |
| Proteobacteria | *Neisseriaceae* | *Neisseria* | 5.859 | 13.570 | 0.000 | 0.000 |
| Actinobacteriota | *Actinomycetaceae* | *Actinomyces* | 5.944 | 13.651 | 0.000 | 0.000 |
| Bacteroidota | *Prevotellaceae* | *Prevotella* | 5.962 | 13.668 | 0.000 | 0.000 |

**Supplementary Table 2:** The Amplicon Sequence Variants (ASV) denoted at the Phylum, Family and Genus-level identified by *edgeR* analysis to be differentially abundant between the FGID patients with or without self-reported non celiac wheat sensitivity (SR-NCWS) (Figure 4., B). The log2-fold change (logFC), log-counts per million reads (logCPM), P and FDR values for those taxa are also shown. Those genera with negative logFC values were found in greater relative abundances for FGID patients without SR-NCWS, while the Genus with positive logFC values were found in greater relative abundances in the FGID patients with SR-NCWS.

| **Phylum** | **Family** | **Genus** | **logFC** | **logCPM** | **PValue** | **FDR** |
| --- | --- | --- | --- | --- | --- | --- |
| Firmicutes | *Streptococcaceae* | *Streptococcus* | -5.409 | 12.515 | 0.000 | 0.000 |
| Firmicutes | *Anaerovoracaceae* | *[Eubacterium]_nodatum_group* | -5.289 | 12.407 | 0.000 | 0.000 |
| Proteobacteria | *Pasteurellaceae* | *Haemophilus* | -4.961 | 12.114 | 0.000 | 0.000 |
| Firmicutes | *Streptococcaceae* | *Streptococcus* | -4.852 | 13.423 | 0.000 | 0.000 |
| Firmicutes | *Streptococcaceae* | *Streptococcus* | -4.401 | 11.632 | 0.000 | 0.000 |
| Firmicutes | *Gemellaceae* | *Gemella* | -4.335 | 11.578 | 0.000 | 0.000 |
| Bacteroidota | *Prevotellaceae* | *Prevotella* | -4.272 | 11.525 | 0.000 | 0.000 |
| Bacteroidota | *Muribaculaceae* | *Muribaculaceae* | -3.402 | 10.852 | 0.000 | 0.000 |
| Bacteroidota | *Prevotellaceae* | *Prevotella* | -3.382 | 10.838 | 0.000 | 0.000 |
| Firmicutes | *Veillonellaceae* | *Veillonella* | -3.203 | 13.005 | 0.001 | 0.008 |
| Firmicutes | *Streptococcaceae* | *Streptococcus* | -3.012 | 10.586 | 0.000 | 0.001 |
| Firmicutes | *Peptostreptococcaceae* | *Peptostreptococcus* | -2.953 | 12.979 | 0.002 | 0.009 |
| Firmicutes | *Streptococcaceae* | *Streptococcus* | -2.873 | 11.258 | 0.001 | 0.007 |
| Firmicutes | *Streptococcaceae* | *Streptococcus* | -2.789 | 11.206 | 0.000 | 0.003 |
| Proteobacteria | *Enterobacteriaceae* | *Escherichia-Shigella* | -2.769 | 10.429 | 0.000 | 0.002 |
| Bacteroidota | *Prevotellaceae* | *Prevotellaceae_UCG-001* | -2.684 | 10.376 | 0.000 | 0.003 |
| Firmicutes | *Clostridiaceae* | *Clostridium_sensu_stricto_1* | 2.608 | 10.926 | 0.002 | 0.009 |
| Proteobacteria | *Pseudomonadaceae* | *Pseudomonas* | 2.799 | 10.405 | 0.000 | 0.001 |
| Firmicutes | *Streptococcaceae* | *Streptococcus* | 3.120 | 11.700 | 0.001 | 0.004 |
| Firmicutes | *Lachnospiraceae* | *GCA-900066575* | 3.169 | 11.757 | 0.001 | 0.005 |
| Firmicutes | *Erysipelotrichaceae* | *Faecalibaculum* | 3.261 | 10.703 | 0.000 | 0.000 |
| Fusobacteriota | *Leptotrichiaceae* | *uncultured* | 3.518 | 10.882 | 0.000 | 0.000 |
| Firmicutes | *Veillonellaceae* | *Veillonella* | 3.634 | 11.396 | 0.000 | 0.000 |
| Actinobacteriota | *Actinomycetaceae* | *Actinomyces* | 3.850 | 11.129 | 0.000 | 0.000 |
| Bacteroidota | *Prevotellaceae* | *Prevotella* | 3.960 | 11.214 | 0.000 | 0.000 |
| Fusobacteriota | *Fusobacteriaceae* | *Fusobacterium* | 4.250 | 11.444 | 0.000 | 0.000 |
| Bacteroidota | *Prevotellaceae* | *Prevotella* | 5.017 | 14.622 | 0.000 | 0.001 |
| Bacteroidota | *Prevotellaceae* | *Prevotella* | 5.130 | 12.968 | 0.000 | 0.000 |
| Bacteroidota | *Prevotellaceae* | *Alloprevotella* | 5.777 | 12.779 | 0.000 | 0.000 |

**Supplementary Table 3:** List of contaminant ASVs (using SILVA_138 99% reference database) generated by the phyloseq “prune_taxa” function. Any ASV observed at a relative abundance greater than 1% on average across 20 reagent controls was included. This criterion ensured all significant contaminants could be removed from the patient samples.

| **ASVs** | **Taxonomy** |
| --- | --- |
| 814cbd873cca731f79b32f02e6cb6550 | d__Bacteria; p__Actinobacteriota; c__Actinobacteria; o__Micrococcales f__Microbacteriaceae; g__Leifsonia;NA |
| 5cd53604a8fef3899e8b1881278ab7ee | d__Bacteria; p__Actinobacteriota; c__Actinobacteria; o__Corynebacteriales f__Corynebacteriaceae; g__Corynebacterium; s__Corynebacterium_aurimucosum |
| bf86bd401f319ddf1cea03466ab3d645 | d__Bacteria; p__Proteobacteria; c__Gammaproteobacteria; o__Burkholderiales f__Comamonadaceae; g__Acidovorax;NA |
| 7244e3c017ce480036880d30250dd471 | d__Bacteria; p__Proteobacteria; c__Gammaproteobacteria; o__Burkholderiales f__Comamonadaceae; g__Comamonas; s__Comamonas_testosteroni |
| 23c12a5ee59b5ee2e7f7660429590b02 | d__Bacteria; p__Proteobacteria; c__Gammaproteobacteria; o__Burkholderiales f__Comamonadaceae; g__Delftia;NA |
| 534fffa65be1aa048d257bd5e1f491e5 | d__Bacteria; p__Proteobacteria; c__Gammaproteobacteria; o__Burkholderiales f__Comamonadaceae; g__Delftia;NA |
| a890f5bedd05f3b2282ad20324166345 | d__Bacteria; p__Proteobacteria; c__Gammaproteobacteria; o__Burkholderiales f__Comamonadaceae; g__Comamonas;NA |
| eaeff860e9316b262edc2f2af44d4edd | d__Bacteria; p__Proteobacteria; c__Gammaproteobacteria; o__Pseudomonadales f__Pseudomonadaceae; g__Pseudomonas;NA |
| a59ecbeff9794b0fc6a51c831a7dfe81 | d__Bacteria; p__Proteobacteria; c__Gammaproteobacteria; o__Burkholderiales f__Comamonadaceae; g__Schlegelella; s__uncultured_bacterium |
| 5525b6511c1d4fd4f8892ee54472e546 | d__Bacteria; p__Proteobacteria; c__Gammaproteobacteria; o__Burkholderiales f__Oxalobacteraceae; g__Massilia;NA |
| 99f5559d291feb4b99c2c2269c881c43 | d__Bacteria; p__Proteobacteria; c__Gammaproteobacteria; o__Burkholderiales f__Oxalobacteraceae; g__Massilia;NA |
| 4a027664246839a242cdc1e23d2168fa | d__Bacteria; p__Proteobacteria; c__Gammaproteobacteria; o__Burkholderiales f__Alcaligenaceae;NA;NA |
| 6304e1d5dbe219667557f5bd13d69c8e | d__Bacteria; p__Firmicutes; c__Bacilli; o__Staphylococcales f__Staphylococcaceae; g__Staphylococcus; s__Staphylococcus_hominis |
| 0795928f3d2bef677e7c910ed1ddaed6 | d__Bacteria; p__Firmicutes; c__Clostridia; o__Lachnospirales f__Lachnospiraceae; g__[Eubacterium]_xylanophilum_group; s__uncultured_bacterium |
| 00eee9faf68bec9994c97c176dcd8f5a | d__Bacteria; p__Firmicutes; c__Bacilli; o__Lactobacillales f__Enterococcaceae; g__Enterococcus; s__Enterococcus_faecalis |
| f887075289522ed7eb4d7deee98231a1 | d__Bacteria; p__Firmicutes; c__Bacilli; o__Lactobacillales f__Enterococcaceae; g__Enterococcus; s__Enterococcus_faecalis |
| 70c8a8ddfd6de2e3b7a387261780a9ac | d__Bacteria; p__Firmicutes; c__Bacilli; o__Paenibacillales f__Paenibacillaceae; g__Paenibacillus;NA |

**Supplementary Materials and Methods:**

**DNA Extraction, library preparation and sequencing**

The protocols used here have been described in detail by Shanahan et al^1^. Briefly, total biopsy DNA is extracted using a repeated bead beating and automated column-based purification, quantified using a dye-based system, and stored at -80^o^C. A set of reagent controls, to which no additional tissue or DNA was added were also prepared. The preparations of the biopsy DNA (or control samples) were used as the template to produce dual-indexed bar-coded PCR amplicons of the V6-V8 hypervariable region of 16S rRNA gene using primers 917F (GAATTGRCGGGGRCC; Bacteria-Domain specific) and 1392R (ACGGGCGGTGWGTRC; Universal). The libraries were sequenced on an Illumina MiSeq using the MiSeq Reagent Kit v3 (2x 300bp), using facilities provided by the Australian Centre for Ecogenomics ([www.ecogenomic.org](http://www.ecogenomic.org)).

**Data processing**

The adapter sequences and tags were trimmed with Cutadapt, and the DADA2 algorithm^2, 3^ embedded within Quantitative Insights into Microbial Ecology version 2 (QIIME2 version 2021.4) was used according to the developer’s recommendations^4, 5^ for sequence quality checking and removal of chimeric sequences, merging of paired-end reads, and generation of amplicon sequence variants (ASVs). The SILVA_138 99% reference database (https://docs.qiime2.org/2021.4/data-resources/) was used to train the feature classifiers and produce taxonomic assignment to the ASVs. Unclassified reads were excluded from the analysis. Given the relatively low microbial density and diversity of these communities, the datasets were further processed to remove contaminant reads (those identified in reagent control samples) as described by Shanahan et al.^1^ and listed in Table S3. Only those datasets from subjects that produced >500 reads after filtering to remove taxa represented at ≤0.05% relative abundance were included in the analyses. These data were subjected to Total Sum Scaling prior to beta-diversity analyses.

**References:**

1. Shanahan ER, Shah A, Koloski N, et al. Influence of cigarette smoking on the human duodenal mucosa-associated microbiota. Microbiome 2018;6:150.

2. Mandal S, Van Treuren W, White RA, et al. Analysis of composition of microbiomes: a novel method for studying microbial composition. Microb Ecol Health Dis 2015;26:27663.

3. Callahan BJ, McMurdie PJ, Rosen MJ, et al. DADA2: High-resolution sample inference from Illumina amplicon data. Nat Methods 2016;13:581-3.

4. Caporaso JG, Kuczynski J, Stombaugh J, et al. QIIME allows analysis of high-throughput community sequencing data. Nat Methods 2010;7:335-6.

5. Hall M, Beiko RG. 16S rRNA Gene Analysis with QIIME2. Methods Mol Biol 2018;1849:113-129.
